# A baseline epidemiological study of the co-infection of enteric protozoans with human immunodeficiency virus among men who have sex with men from Northeast China

**DOI:** 10.1371/journal.pntd.0010712

**Published:** 2022-09-06

**Authors:** Wei Zhao, Lan Yao, Min Zhuang, Yuan-Long Lin, Xiao-Hong Chen, Li Wang, Bo Song, Ya-Shuang Zhao, Yun Xiao, Feng-Min Zhang, Fu-Xiang Wang, Hong Ling

**Affiliations:** 1 Department of Microbiology, School of Basic Medicine, Wu Lien-Teh Institute, Harbin Medical University, Heilongjiang Provincial Key Laboratory of Infection and Immunity, Key Laboratory of Pathogen Biology, Harbin, China; 2 Department of Parasitology, School of Basic Medicine, Wenzhou Medical University, Wenzhou, China; 3 Department of Parasitology, School of Basic Medicine, Harbin Medical University, Harbin, China; 4 Shenzhen Key Laboratory of Pathogen and Immunity, National Clinical Research Center for Infectious Disease, State Key Discipline of Infectious Disease, Shenzhen Third People’s Hospital, Second Hospital Affiliated to Southern University of Science and Technology, Shenzhen, China; 5 Department of Infectious Diseases, Fourth Affiliated Hospital of Harbin Medical University, Harbin, China; 6 The Heilongjiang province hospital, Harbin, China; 7 Department of Epidemiology, Public Health College, Harbin Medical University, Harbin, China; 8 College of Bioinformatics Science and Technology, Harbin Medical University, Harbin, China; National University of Singapore, SINGAPORE

## Abstract

**Background:**

Human immunodeficiency virus (HIV) and enteric parasite co-infection not only aggravates the clinical symptoms of parasites but also accelerates acquired immunodeficiency syndrome (AIDS) progression. However, co-infection research on men who have sex with men (MSM), the predominant high-risk population of HIV/AIDS in China, is still limited. In this study, we investigated the epidemiology of enteric parasites, risk factors, and associations with clinical significance in an MSM HIV/AIDS population in Heilongjiang Province, northeast China.

**Methods:**

We recruited 308 MSMs HIV/AIDS patients and 199 HIV-negative individuals in two designated AIDS hospitals in Heilongjiang between April 2016 and July 2017. Fresh stool samples were collected. DNA extraction, molecular identification, and genotyping of *Cryptosporidium* species, *Entamoeba histolytica*, *Cyclospora cayetanensis*, *Enterocytozoon bieneusi*, and *Blastocystis hominis* were performed. Fourteen diarrhea-related pathogens were examined to exclude the influence of other bacterial pathogens on diarrhea incidence.

**Results:**

31.5% of MSM HIV/AIDS participants were infected with at least one parasite species, a significantly higher proportion than that found in the HIV-negative individuals (2.5%). *E*. *bieneusi* presented the highest prevalence, followed by *B*. *hominis*, *E*. *histolytica*, *Cryptosporidium* spp., and *C*. *cayetanensis*. Warm seasons were the risk factor for parasitic infections in this population [odds ratio (OR) = 2.6, 95% CI: 1.47–4.57]. In addition, these individuals showed a higher proportion (35.8%) of present diarrhea (PD) compared with men who have sex with women (MSW) with HIV/AIDS (16.7%). The infection proportions of both *Cryptosporidium* spp. and *E*. *histolytica* were significantly higher in the PD. *E*. *bieneusi* infection was more prevalent in the historic diarrhea (HD) group. CD4^+^ T cell counts in the MSM patients with the above three parasites were significantly lower. New species and genotypes were found, and MSM patients had a wider range of species or genotypes.

**Conclusions:**

Enteric parasitic infection was prevalent in the MSM HIV/AIDS population, especially in patients with present diarrhea during warm seasons. *E*. *histolytica* and *B*. *hominis* should also be considered high-risk parasites for opportunistic infections in AIDS patients in addition to *Cryptosporidium* spp.

## Author summary

Human immunodeficiency virus (HIV) and enteric parasite co-infection not only aggravates the clinical symptoms of parasites but also accelerates acquired immunodeficiency syndrome (AIDS) progression. In China, despite a large number of HIV-infected people, the data regarding co-infection with enteric intracellular parasites in this population is still sparse. In the present study, we investigated the epidemiology of *Cryptosporidium* spp., *Enterocytozoon bieneusi*, *Blastocystis hominis*, *Entamoeba histolytica* and *Cyclospora cayetanensis* in 384 HIV/AIDS including 308 MSM HIV/AIDS populations in two designated AIDS hospitals in Heilongjiang Province, North-East China between April 2016 and July 2017 by polymerase chain reaction. We also try to track the possible sources, risk factors, and any associations, with clinical significance, of human parasitic infections. Such knowledge will provide insights into prognosis, treatment, and preventive strategies against infections with such pathogens. The findings in this study have important implications for public health, the control of AIDS progression and control of parasitic infections.

## Introduction

Human immunodeficiency virus (HIV) and acquired immunodeficiency syndrome (AIDS), caused by HIV infection, remain significant public health challenges. HIV infection leads to severe dysfunction of both adaptive and innate immune responses and results in severe opportunistic infections [1]. HIV-positive hosts and AIDS patients (HIV/AIDS) are at a higher risk for opportunistic infections, including enteric parasites, particularly when combined with impaired or deficient T cell function [2]. Moreover, HIV infection may negatively impact the natural evolution of certain parasitic infections, lead to more serious clinical symptoms, and complicate the treatment of both diseases [3]. Conversely, the mucosal damage and changes in the immunological mini-environment caused by some parasitic infections may increase susceptibility to HIV infections, facilitate HIV replication, and accelerate disease progression [4]. This field has gradually attracted more attention due to the adverse impacts of co-infections in HIV/AIDS individuals. However, the epidemiological characteristics of co-infection remain poorly understood, as there are many parasite species, and the prevalence varies in different parts of the world [5, 6].

China has a large number of HIV-infected individuals, and HIV transmission patterns have changed dramatically in recent years. By the end of 2020, China had approximately 1053 thousands people with HIV/AIDS, and there were an estimated 34,512 new HIV infections in 2017, and 25.5% of these were men who have sex with men (MSM) [7,8]. In addition, it was reported that the number of university students infected with HIV increased by 35% annually from 2011 to 2015, possibly due to an increase in MSM students [9]. Growing numbers of MSM individuals in China and the rise of HIV infection in the population have become a significant challenge for HIV/AIDS prevention [7].

As a high-risk population of HIV, MSM individuals also have an increased risk of contracting sexually transmissible enteric infections (STEIs), and the widespread distribution of STEI outbreaks in MSM warrants public health attention [10]. Investigators have found that the prevalence of enteric parasites reached 52–59% in the MSM populations of Scotland, Australia, and the USA [11–13]. Of the enteric parasites found in HIV/AIDS individuals, some genera of protozoa, such as *Cryptosporidium*, *Cyclospora*, *Isospora*, *Microsporidia* (phylogenetically related to fungi), and *Blastocystis*, are prevalent worldwide [5, 6].

In China, despite a large number of HIV-infected people, the data regarding co-infection with enteric intracellular parasites in this population is still sparse. To date, several reports have focused on HIV and intestinal parasites in middle and southern China [5, 14–16]. However, molecular detection methods were used in no more than two of those reports [16, 17]. Furthermore, concerning parasitic intestinal infections in the MSM population of mainland China, only one study has been published, and the seroprevalence of *E*. *histolytica* was found to be 41.1% in Beijing and Tianjin [18].

Epidemiological investigations have shown that some enteric pathogens of humans (including *Cryptosporidium* spp. *Enterocytozoon bieneusi* and *Blastocystis* spp.) are widespread in nature and can infect multiple animals, including farm, household, and wild animals. In addition, there are multiple zoonotic species/genotypes of these pathogens [19–21]. Therefore, we propose that the scale of infection by enteric intracellular parasites in HIV/AIDS individuals has been underestimated in China. More importantly, as enteric parasitic infections are associated with either severe diarrhea or worsening severity of other symptoms in HIV/AIDS individuals, co-infection in this population poses a significant threat if awareness of these dynamics continues to be neglected.

In Heilongjiang Province, northeast China, MSM HIV/AIDS individuals account for >75% of all those infected with HIV [22] and where enteric parasites in local animals are widely distributed (S1–S3 Tables). Nevertheless, the co-infection of enteric intracellular parasites with the human immunodeficiency virus (HIV) among homosexuals in northeast China remains poorly defined. Therefore, the present study aimed to investigate the epidemiology of five enteric parasites and evaluate risk factors and associations with clinical significance in an HIV/AIDS population, especially for MSM in Heilongjiang Province, northeast China.

## Materials and methods

### Ethics approval and consent to participate

Approvals for these studies were obtained from the Ethics Committees of the Fourth Affiliated Hospital of Harbin Medical University and the Harbin Infectious Disease Hospital (SCILLSC-2017-01). Written informed consent was signed by each participant (for the 17-year-old participants, the consent forms were obtained from their parents or guardian) after they or their parents or guardian were informed of the purposes and procedures of the study.

### Study area

**Fig 1:**
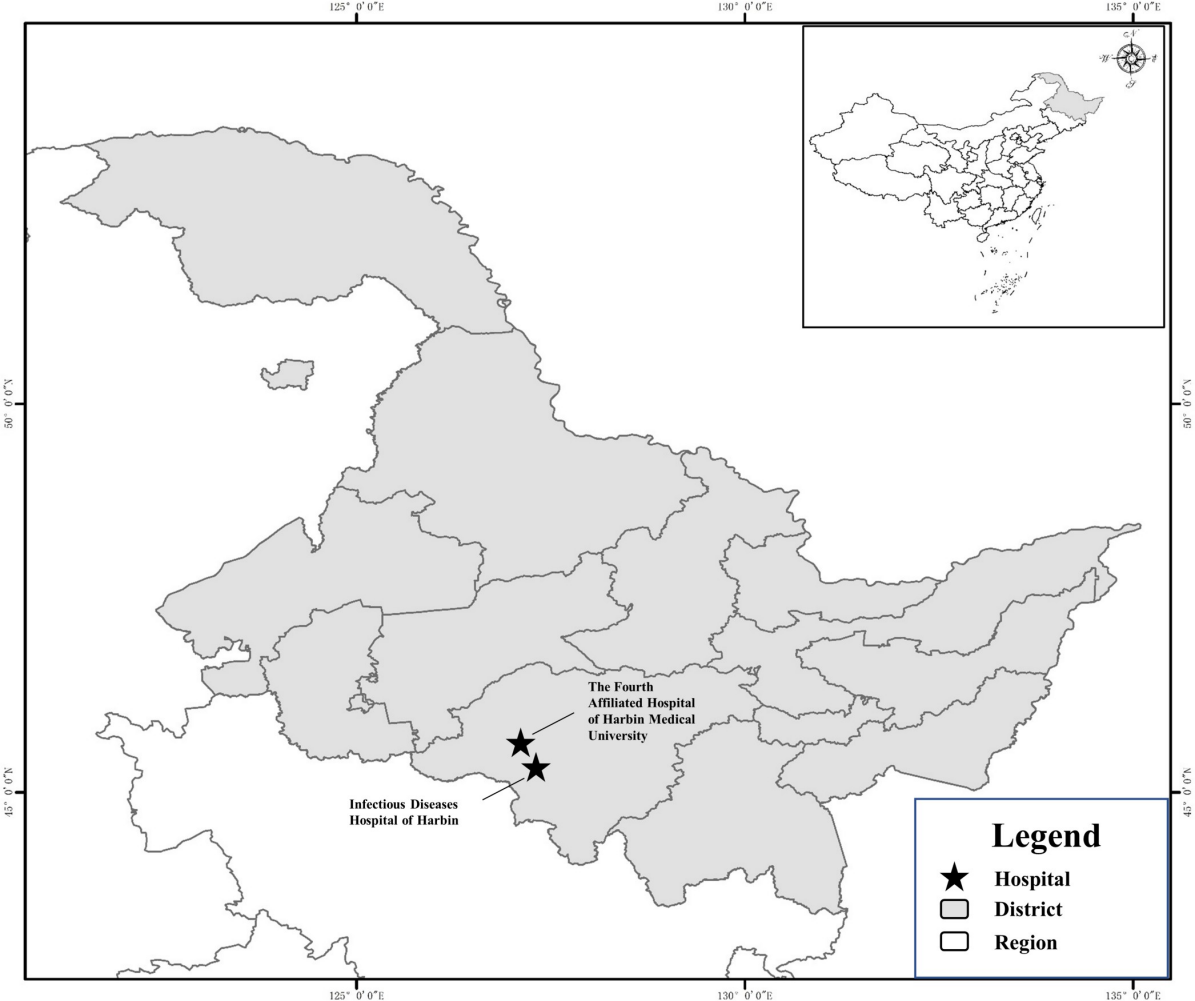
Map of the two hospitals where samples were collected location in Heilongjiang Province, China. The map was originally designed using the software ArcGIS 10.4. The original vector diagram was adopted from National Geomatics Center of China (http://www.ngcc.cn). The map has been modified and assembled according to permission and attribution guidelines using Microsoft PowerPoint 2003 and Adobe Photoshop CS6.

The study was conducted in two public designated AIDS hospitals in northeast China: the Fourth Affiliated Hospital of Harbin Medical University and the Harbin Infectious Disease Hospital. These hospitals are located in the Xiangfang and Nangang districts of Harbin City, respectively (Fig 1).

### Study population

The study population consisted of patients with HIV/AIDS, who underwent outpatient examinations or hospitalization at the two designated AIDS treatment hospitals in Heilongjiang Province. In addition, the age-matched, HIV-negative, healthy individuals were recruited from the medical examination center of the Fourth Affiliated Hospital of Harbin Medical University.

### Sampling method and data collection

Between April 2016 and August 2017, fecal samples were collected from the patients with HIV/AIDS. The patients or their parents/guardians who provided consent on behalf of their children were trained in the relevant guidelines by the staff and were provided with a labeled plastic fecal collector marked with the date of collection and patient identity information (age and sex). After collection, the samples were stored at 4°C.

Each HIV-positive participant was interviewed using a standardized questionnaire to collect information regarding sociodemographic characteristics, living habits, animal exposure history, medication history (including the use of antiretroviral drugs and antibiotics), and recent clinical manifestations. Present diarrhea (PD) was defined as more than three episodes of watery stools or loose stools per day at the sampling time. Historic diarrhea (HD) was defined as having a history of diarrhea (two or more instances of watery stools or loose stools per day, more than five days within the month before sampling) but no diarrhea at the time of sampling. The HIV infection and AIDS stages of participants were determined according to World Health Organization (WHO) guidelines [23].

Moreover, we collected the CD4^+^ T cell counts, HIV viral load (VL), and routine blood parameters, including white blood cell (WBC), neutrophil (NEUT), lymphocyte (LYMPH), red blood cell (RBC), hemoglobin (HGB), monocyte (MONO), eosinophil (EOSO) and basophil (BASO) counts, obtained from the sampling hospital to evaluate changes in the parameters in the presence of parasitic infections.

### Sample size determination

A total of 601 fecal samples were collected from humans in the two hospitals in Heilongjiang Province, China, including 402 HIV-positive participants drawn from various regions of Heilongjiang Province and diagnosed in two designated AIDS hospitals in Harbin, the Fourth Affiliated Hospital of Harbin Medical University and the Harbin Infectious Disease Hospital and 199 HIV-negative healthy individuals recruited from the medical examination center of the Fourth Affiliated Hospital of Harbin Medical University.

### Inclusion/exclusion criteria

Doctors communicated and provided consulting services for voluntary participation in the survey and signed written informed consent forms. The participants had not undergone an antiparasitic treatment. Fecal samples of the HIV-positive participants were used for parasitological analysis only if they were coupled with the questionnaire and appropriate clinical data.

### DNA extraction and PCR amplification

Genomic DNA was extracted from 180–200 mg of the fecal specimen using a QIAamp DNA Stool Mini Kit (QIAgen, Hilden, Germany), following the manufacturer’s guidelines. DNA was finally eluted in 200 μL of AE buffer and stored at –20°C until further use in PCR analysis. The presence of *Cryptosporidium*, *E*. *histolytica*, *Cyclospora*, *E*. *bieneusi*, and *Blastocystis* was examined using nested PCR amplification based on the methods described elsewhere [24–28]. To further identify the genotypes of *E*. *bieneusi* isolates, a 389-bp fragment of rDNA, including part of the small subunit (SSU), internal transcribed spacer (ITS) region, and part of the large subunit (LSU), was amplified using a new nested PCR [29]. Subtypes of *Cryptosporidium* isolates were identified using an approximately 800–850-bp fragment of the *gp60* gene [30]. S4 and S5 Tables present the primers used to identify the parasites and the genotypes of *E*. *bieneusi* and subtypes of *Cryptosporidium* and the conditions of the PCR experiment, respectively (S4 and S5 Tables). TaKaRa Taq DNA Polymerase (TaKaRa Bio Inc., Tokyo, Japan) was used in PCR amplification. A negative control without DNA was included in all the tests. Each specimen was analyzed twice, with 2 μL of DNA used as a template for each PCR. All the secondary PCR products were subjected to electrophoresis on a 1.5% agarose gel and visualized by staining the gel with Goldenview.

The presence of 14 diarrhea-associated pathogens, including *Staphylococcus aureus*, *Salmonella*, *Enterobacter sakazakii*, *Yersinia enterocolitica*, *Aeromonas hydrophila*, *Bacillus cereus*, *Listeria monocytogenes*, *Escherichia coli O157*, *Shigella castellani*, *Vibrio cholerae*, *Campylobacter coli*, *Vibrio parahaemolyticus*, *Escherichia coli*, and *Campylobacter jejuni* in the stool samples was examined using a Multiplex PCR diagnostic kit for rapid identification of foodborne pathogenic bacteria (Beijing Applied Biological Technologies Co., Ltd.; Beijing, China).

### Nucleotide sequencing and molecular analysis

All PCR products of the expected size were purified and then sequenced with their respective primers by Sinogeno-max Biotechnology Co. Ltd. (Beijing, China), using a Big Dye Terminator v3.1 Cycle Sequencing Kit (Applied Biosystems, USA) on an ABI PRISM 3730 XL DNA Analyzer. The accuracy of nucleotide sequences was confirmed by sequencing from both ends of the product and by sequencing additional PCR products when mutations were detected.

All the sequences obtained in this study were subjected to BLAST searches (http://www.ncbi.nlm.nih.gov/blast/) and aligned with reference sequences to determine the species and genotypes/subtypes using Clustal X 1.83 (http://www.clustal.org/). Novel genotypes of *E*. *bieneusi* were identified if the sequences were not identical to known genotypes in the GenBank database, according to the established nomenclature system [31].

### Statistical analysis

Data entry and analysis were performed using Statistical Package for Social Sciences (SPSS) 19.0 software. The statistical significance of differences in infection proportions was generally evaluated by Pearson’s chi-squared test, while the Fisher’s exact test was used when more than 20% of cells in contingency tables had the expected frequencies of <5. In addition, bivariate and multivariate logistic regression analyses were used to assess the possible risk factors associated with parasitic infections in HIV-positive participants.

The data contained 9 variables, including routine blood parameters (WBC, NEUT, LYMPH, MONO, EOSO, BASO, RBC, and HGB) and CD4^+^ T cell counts, as well as associated parasitic infection status and the AIDS stage of all MSM participants. Outlier values were removed to reduce their influence on the analyses. To generate the heat map of the data, hierarchical clustering was performed with average linkage and Pearson’s correlation. Next, the cluster groups were determined using the dynamic tree cut algorithm [32]. Following clustering, the relationship between the parasitic infection status and the cluster groups was tested using the chi-squared test or Fisher’s exact test. The above data analyses were carried out using R software, version 3.4.1.

The significant level of all the tests was established with a p-value threshold of 0.05. Then, the 95% confidence intervals (95% CI) for prevalence were calculated based on the Poisson distribution.

## Results

### Samples and patient data

**Fig 2:**
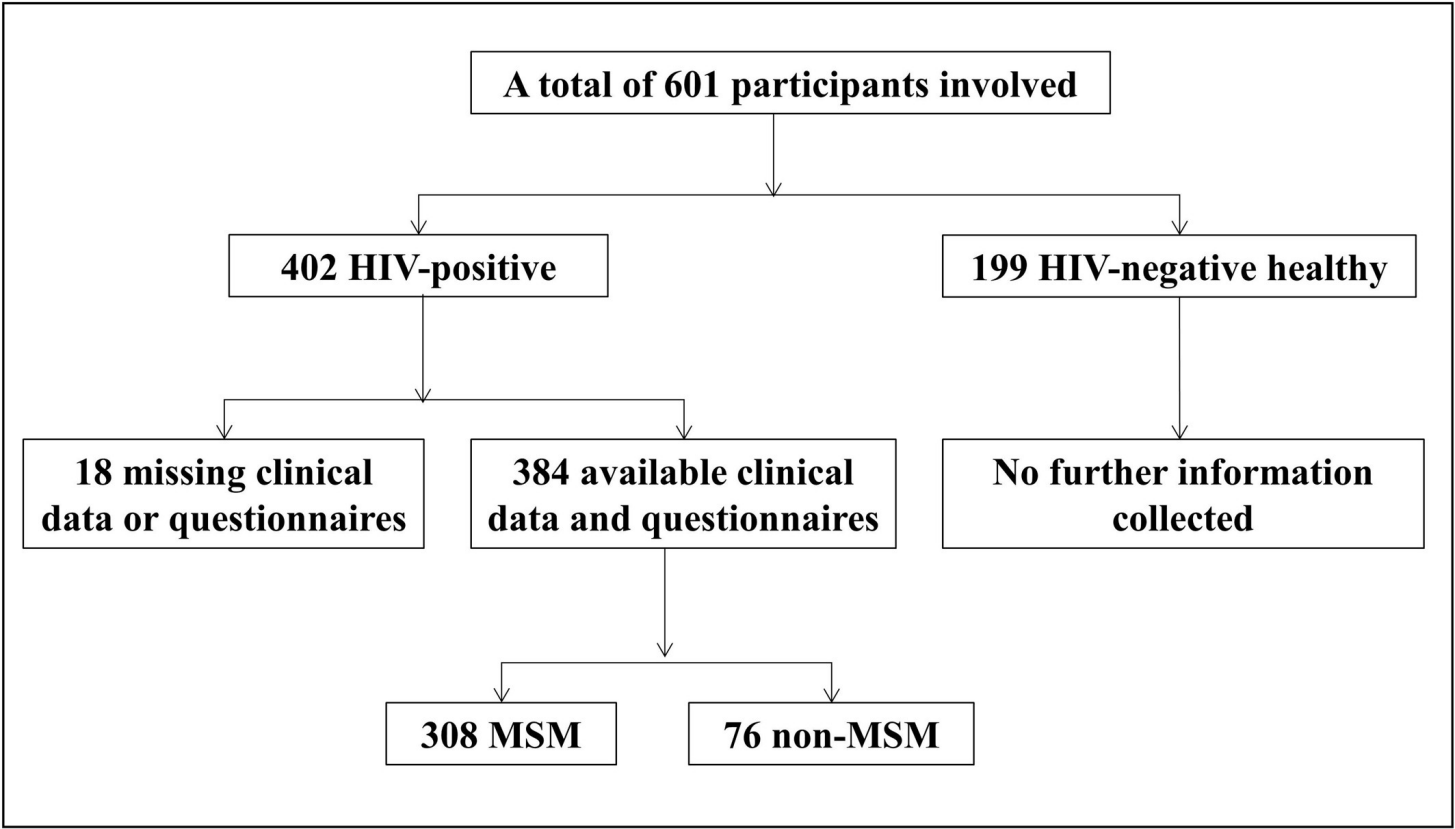
Number of study participants at various stages of recruitment.

Altogether, 384 out of the 402 HIV/AIDS individuals had available clinical data and questionnaires (Fig 2). The average age of HIV/AIDS individuals was 36.9 years, ranging from 17 to 76 years. Among them, 96.1% were male, 66.7% were non-farmers, 66.7% drank boiled water, 30% had a history of contact with animals, 41.1% had an antiretroviral therapy (ART), 65.1% had an antibiotic treatment history, and 46.4% had diarrhea (S6 Table). Of all the subjects, 80.2% were MSM. According to WHO clinical staging guidelines, 118 MSM HIV/AIDS individuals were at stages I and II (I/II), referred to as early-stage, and 190 were at stages III and IV (III/IV), referred to as late-stage. Meanwhile, 150 and 234 the samples were collected in May to October (warm season) and November to April (cold season), respectively (S7 Table). For the 199 HIV-negative individuals, no information was collected except for age; their average age was 35.8 years and ranged from 20 to 62 years.

### The prevalence of enteric parasites in healthy and HIV-infected individuals

Among the 199 HIV-negative individuals, the overall prevalence of *B*. *hominis* and *E*. *bieneusi* was 1.5% (3/199) and 1.0% (2/199), respectively. No other parasites were found in the healthy individuals (Table 1). On the contrary, 31.5% (121/384) of the HIV/AIDS individuals were co-infected with at least one species of parasites. Of the 384 HIV/AIDS patients, *E*. *bieneusi* infection (14.6%) was the most predominant, followed by *B*. *hominis* (8.1%), *E*. *histolytica* (6.3%), and *Cryptosporidium* spp. (3.4%), while *C*. *cayetanensis* was the least prevalent (1.8%) (Table 1). Mixed infections with two species of parasite were found in 10 patients (2.6%). Among these 10 individuals, co-infection with *B*. *hominis* and *E*. *bieneusi* was the most common occurrence (S8 Table).

**Table 1:** Prevalence of five enteric intracellular parasites among HIV-negative and HIV-positive participants with different transmission routes.

Parasites	HIV-negative (n = 199)		HIV-positive (n = 384)		Routes of HIV infection					
					MSM (n = 308)		MSW (n = 31)		Others (n = 45)	
	n	%	n	%	n	%	n	%	n	%
Any of parasites	5	2.5	121	31.5	92	29.9	12	35.5	18	40.0
*E*. *histolytica*	0	/	24	6.3	19	6.2	3	9.7	2	4.0
*E*. *bieneusi*	2	1.0	56	14.6	45	14.6	5	16.1	6	13.3
*Cryptosporidium* spp.	0	/	13	3.4	9	2.9	2	6.5	2	4.0
*C*. *cayetanensis*	0	/	7	1.8	5	1.6	1	3.2	1	2.0
*B*. *hominis*	3	1.5	31	8.1	19	6.2	5	16.1	7	15.6

MSM = men having sex with men. MSW = men having sex with women. Others = sexually transmitted women, non-sexually transmitted individuals, and unknown transmission routes were included

For MSM HIV/AIDS, the overall prevalence of all five parasites was 29.9% (92/308). *E*. *bieneusi* still showed the highest prevalence (14.6%), followed by *B*. *hominis* (6.2%), *E*. *histolytica* (6.2%), *Cryptosporidium* spp. (2.9%), and *C*. *cayetanensis* (1.6%) (Table 1). Surprisingly, the prevalence of *B*. *hominis* in the MSM population (6.2%) was significantly lower than in the men having sex with women MSW group (16.1%) and others (women, non-sexually transmitted individuals and unknown transmission routes) (15.6%) (Table 1).

### Risk factors of parasitic infection in HIV-infected individuals

Proportion of any parasite was not significantly different between age groups. The total positivity rate for intestinal protozoa was lower in males than females with HIV infection, but the difference was not significant (*p* = 0.661). Positivity rates for different parasite species differed between male and female patients, with a significantly higher rate of *B*. *hominis* positivity in females than in males (*p* = 0.027). The total positivity rates for intestinal protozoa (*p* = 0.002), *E*. *bieneusi* (*p* = 0.002) and *B*. *hominis* (*p* = 0.029) in farmers, were significantly higher than those in non-farmers. (S9 Table).

The total positivity rate for protozoa in the group drinking raw water was slightly higher than that in the group drinking boiled water (*p* = 0.187). The positivity rate for *Cryptosporidium* in the group drinking raw water was significantly higher than that in the group drinking boiled water (*p* = 0.028); other intestinal protozoan infections were not related to drinking water. (S9 Table).

The total intestinal protozoan infections in these individuals, as well as infections with protozoa other than *C*. *cayetanensis*, were higher than in those without animal contact, although the differences were not significant. (S9 Table).

The total protozoa positivity rate was significantly higher (*p* < 0.00) for samples collected during the warm season (May to October) than for those collected during the cool season (November to April of the next year) (*p* < 0.001). Except for *Cryptosporidium*, the other four protozoa positivity rates were higher in the warm season than in the cold season, with significant differences between *E*. *histolytica* (*P* = 0.001), *E*. *bieneusi* (*p* < 0.001), and *B*. *hominis* (*p* = 0.005). (S9 Table).

The presence or absence of antibiotics did not significantly affect the overall infection with protozoa; however, the positivity rate for *B*. *hominis* appeared to be somewhat higher in patients taking antibiotics (*p* = 0.058). (S8 Table).

Except for *E*. *bieneusi*, the other four protozoa were positively correlated with the stage of AIDS disease. The rate of positivity for all intestinal protozoa was significantly higher in the III/IV patients than in the I/II patients (*p* < 0.05) except for *E*. *bieneusi*, but the rate of positivity for *E*. *bieneusi* was slightly higher in stage I/II patients (*p* = 0.07). (S9 Table).

There was no difference in the overall positivity rate for intestinal protozoa between the different HIV transmission route groups, and the positivity rate for *B*. *hominis* was significantly lower in the MSM population than in the other two groups (*p* = 0.02). (S9 Table)

### The risk factor of parasitic infection in MSM individuals

Concerning MSM HIV/AIDS, *E*. *histolytica* (*p* < 0.001), *C*. *cayetanensis* (*p* = 0.006), and *B*. *hominis* (*p* < 0.002) were more prevalent in the warm season than in the cold season (Table 2). The infection proportion of any parasite at the late stage (36.4%) was significantly higher than at the early stage (22.6%) (*p* = 0.004) (Table 2). *E*. *histolytica* and *B*. *hominis* were more common in late-stage patients than in early-stage patients (9.5% vs. 0.8%, *p* = 0.005; 8.9% vs. 1.7%, *p* = 0.020, respectively) (Table 2). However, the prevalence of *E*. *bieneus*i in early-stage patients (19.5%) was slightly higher than that in late-stage patients (11.6%) (*p* = 0.081; Table 2). The univariate analysis showed that, apart from seasonality and AIDS stages, CD4^+^ counts were significant predictors (*p* < 0.05) (Table 3). However, multivariable analysis showed that the warm season was the only risk factor for parasitic infection [odds ratio (OR) = 2.6, 95% CI: 1.47–4.57] (Table 3).

**Table 2:** Risk factors associated with enteric parasite infection in MSM HIV-positive participants.

Characteristics		No.	Any of parasites		*E*. *histolytica*		*E*. *bieneusi*		*Cryptosporidium*		*C*. *cayetanensis*		*B*. *hominis*	
			n (%)	p value	n (%)	p value	n (%)	p value	n (%)	p value	n (%)	p value	n (%)	p value
Age														
	≤30	131	36 (27.5)	0.509	8 (6.1)	0.653	19 (14.5)	0.519	3 (2.3)	0.108	2 (1.5)	0.230	6 (4.6)	0.285
	31–50	142	47 (33.1)		10 (7.0)		23 (16.2)		3 (2.1)		2 (0.7)		12 (8.5)	
	>50	35	9 (25.7)		1 (2.9)		3 (8.6)		3 (8.6)		1 (2.9)		1 (2.9)	
Occupation														
	Farmer	86	30 (34.9)		3 (3.5)		16 (18.6)		4 (4.7)		0		7 (8.1)	
	No-farmer	222	62 (27.9)	0.231	16 (7.2)	0.341	29 (13.1)	0.217	5 (2.3)	0.457	5 (2.3)	0.327*	12 (5.4)	0.371
Drinking boiled water														
	Yes	207	58 (28.0)		14 (6.8)		29 (14.0)		4 (1.9)		2 (1.0)		13 (6.3)	
	No	101	34 (33.7)	0.310	5 (5.0)	0.535	16 (15.8)	0.669	5 (5.0)	0.264	3 (3.0)	0.409	6 (5.9)	0.907
Contact with animal														
	Yes	97	33 (34.0)		7 (7.2)		14 (14.4)		4 (4.1)		0		9 (9.3)	
	No	211	59 (28.0)	0.281	12 (5.7)	0.604	31 (14.7)	0.952	5 (2.4)	0.628	5 (2.4)	0.330*	10 (4.7)	0.124
Season (the samples and the patient data collected dates)														
	Nov.-Apr	197	43 (21.8)		5 (2.5)		27 (13.7)		7 (3.6)		0		6 (3.0)	
	May.-Oct	111	**49 (44.1)**	0.000	**14 (12.6)**	0.000	18 (16.2)	0.549	2 (1.8)	0.600	**5 (4.5)**	0.006	**13 (11.7)**	0.002
Antibiotics														
	Yes	190	58 (30.5)	0.750	13 (6.8)	0.533	26 (13.7)	0.559	6 (3.2)	1.000	2 (1.1)	0.588	15 (7.9)	0.176
	No	118	34 (28.8)		6 (5.1)		19 (16.1)		3 (2.5)		3 (2.5)		4 (3.4)	
AIDS stages														
	I-II	118	27 (22.6)	0.035	1 (0.8)	0.005	23 (19.5)	0.081	1 (0.8)	0.161	0	0.161*	2 (1.7)	0.020
	III-IV	190	**65 (36.4)**		**18 (9.5)**		22 (11.6)		8 (4.2)		5 (2.6)		**17 (8.9)**	
Total		308	92 (29.9)		19 (6.2)		45 (14.6)		9 (2.9)		5 (1.6)		19 (6.2)	

*Fisher’s Exact Test. Bold = the values statistically significant higher than that in the same group were shown in bold.

**Table 3:** Univariate and Multivariable analysis of the potential risk factors of parasites infection in MSM HIV/AIDS participants.

Characteristics	n	Infection n (%)	Univariate analysis		Multivariable analysis	
			p value	Crude OR (95% CI)	p value	Adjusted OR (95% CI)
Age						
≤30	131	36 (27.5)	0.84	1.1 (0.47–2.56)	0.59	1.3 (0.51–3.29)
31–50	142	47 (33.1)	0.40	1.4 (0.62–3.29)	0.37	1.5 (0.61–3.83)
>50	35	9 (25.7)	1.00		1.00	
Residence						
Farmer	86	30 (34.9)	0.23	1.4 (0.81–2.35)	0.204	1.5 (0.81–2.68)
Non-farmer	222	62 (27.9)	1.00		1.00	
Drinking boiled water						
Yes	207	58 (28.0)	0.31	1.3 (0.78–2.18)	0.73	1.1 (0.63–1.95)
No	101	34 (33.7)	1.00		1.00	
Contact with animals						
Yes	97	33 (34.0)	0.28	1.3 (0.79–2.22)	0.99	1.0 (0.56–1.77)
No	211	59 (28.0)	1.00		1.00	
Season						
May-Oct.	111	**49 (45**.**1)**	0.000	2.8 (1.71–4.69)	0.001	2.6 (1.47–4.57)
Nov.-Apr.	197	43 (21.8)	1.00		1.00	
Antibiotics						
Yes	190	58 (30.5)	0.75	1.1 (0.66–1.80)	0.75	0.91 (0.51–1.61)
No	118	34 (28.2)	1.00		1.00	
AIDS stages						
I-II	118	27 (22.9)	0.041	0.6 (0.34–0.96)	0.65	0.8 (0.36–1.92)
III-IV	190	**65 (34**.**2)**	1.00		1.00	
CD4^+^ counts						
<150	121	**47 (38.8)**	0.031	2.0 (1.1–3.6)	0.30	1.5 (0.68–3.47)
150–350	97	23 (23.7)	0.91	1.0 (0.5–1.9)	0.55	0.8 (0.38–1.66)
>350	90	22 (24.4)	1.00		1.00	

Bold = the values statistically significant higher than that in the same group were shown in bold. OR = odds ratio.

### Parasitic infection and diarrhea

To exclude the influence of bacterial pathogens on diarrhea, we examined 14 diarrhea-associated pathogens. We found that three parasite-infected patients were co-infected with *Yersinia enterocolitica*, and one was co-infected with *Vibrio parahaemolyticus*. These four cases were removed from the diarrhea group in the following analysis of the association of the infection with diarrhea. In MSM HIV/AIDS population, the overall prevalence of parasites in diarrhea patients was higher than that in non-diarrhea patients (ND) (38.8% vs. 18.9%, *p* < 0.001), and both PD and HD were higher than that in ND (44.4% vs 18.9%, *p* < 0.001 and 35.7% vs 18.9%, *p* = 0.003, respectively) (Table 4). Meanwhile, there is statistical different of the overall prevalence of parasites among the PD, HD and ND groups (44.4% vs 35.7% vs 18.9%, *p* < 0.001) (Table 4). In particular, the infection proportion of *E*. *bieneusi*, *E*. *histolytica*, and *Cryptosporidium* spp. in diarrhea patients was higher than in non-diarrhea subjects, with only the difference for *E bieneusi* being statistically significant (17.8% vs. 9.5%, *p* = 0.034) (Table 4). Furthermore, the infection proportions of both *Cryptosporidium* spp. and *E*. *histolytica* were slightly higher in PD individuals than in HD individuals (8.9% and 15.5% vs. 0.6% and 3.6%, respectively) (Table 4). On the other hand, *E*. *bieneusi* infection was significantly more prevalent in the HD group than in the PD group (21.4% vs. 11.1%) (Table 4). *B*. *hominis* and *C*. *cayetanensis* did not show any difference in the infection proportion between diarrhea and non-diarrhea groups (Tables 4 and S10).

**Table 4:** Parasite infection proportion in MSM HIV-positive participants with different diarrhea status.

Diarrhea status ^#^		n	Any of parasites		*E*. *histolytica*		*E*. *bieneusi*		*Cryptosporidium*		*C*. *cayetanensis*		*B*. *hominis*	
			n (%)	p value	n (%)	p value	n (%)	p value	n (%)	p value	n (%)	p value	n (%)	p value
Yes	PD^a^	45	**20 (44.4)**	0.000	**7 (15.5)**	0.003	5 (11.1)	0.742	**4 (8**.**9)**	0.007	**1 (2.2)**	0.752	3 (6.7)	1.000
	HD^b^	84	30 (35.7)	0.003	4 (4.8)	0.902	**18 (21.4)**	0.009	1 (1.2)	1.000	1 (1.2)	0.309	**6 (7.1)**	1.000
	Subtotal^c^	129	50 (38.8)	0.332	11 (8.5)	0.078	23 (17.8)	0.145	5 (3.9)	0.093	2 (1.6)	1.000	9 (7.0)	1.000
No	ND^d^	169	32 (18.9)	0.000	6 (3.6)	0.066	16 (9.5)	0.034	1 (0.6)	0.113	2 (1.2)	1.000	7 (4.1)	0.282
Total^e^		308	92 (29.9)	0.000	19 (6.2)	0.008	45 (14.6)	0.026	9 (2.9)	0.006	5 (1.6)	1.000	19 (6.2)	0.591

PD = present diarrhea. HD = historic diarrhea, as described in the methods. ND = non-diarrhea. Bold = the values statistically significant higher than that in the same group were shown in bold.

^#^Parasite infection identified as a single infection.

^a^p value = PD vs ND.

^b^p value = HD vs ND.

^c^p value = PD vs HD.

^d^p value = Yes vs No.

^e^p value = PD vs HD vs ND.

### Parasitic infection and hemogram parameters

**Fig 3:**
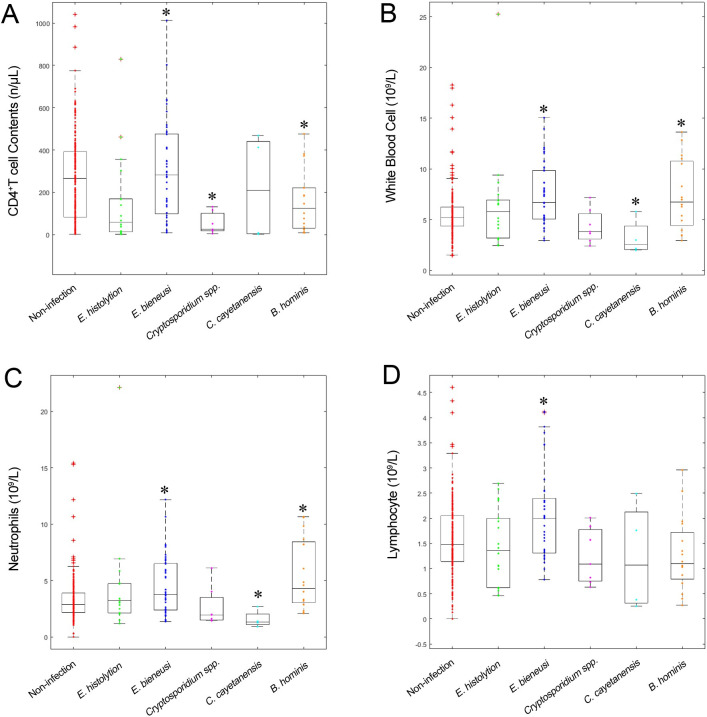
CD4^+^ T cell counts and hemogram parameters in different parasite-infection and non-infection groups of the MSM HIV/AIDS population. CD4^+^ T cell counts (A), WBC (B), NEUT (C) and LYMPH (D) in different parasite-infection and non-infection groups.* indicates statistical significant difference between the population with parasite infections versus the one without it.

To eliminate the influence of mixed infections with different parasites, we included only cases of single infection (n = 302). CD4^+^ T cell, WBC, NEUT, and LYMPH counts were significantly different between individuals with and without enteric parasite infections (Table 5 and Fig 3). CD4^+^ T cell counts were lower in patients infected with *Cryptosporidium* spp. (52.1 ± 50.9), *E*. *histolytica* (153.1 ± 215.8), or *B*. *hominis* (157.4 ± 146.6) than in the non-infected group (266.1 ± 201.3) (*p* < 0.001, *p* = 0.002, and *p* = 0.006, respectively). Furthermore, the CD4^+^ T cell count was below 150 cells/μL in all nine *Cryptosporidium*-infected MSM HIV/AIDS individuals (S11 Table). There were no significant differences in routine hemogram parameters between *Cryptosporidium*-infected and non-infected MSM HIV/AIDS individuals. WBC and most of its components, except basophil (BASO), were elevated in *E*. *bieneusi*-infected patients.

**Table 5:** The hemogram parameters and CD4^+^ T cell counts of MSM HIV-positive participants with parasitic infection (Mean ± SD).

Parameters	Non-infection	*Cryptosporidium*	*E*. *histolytica*	*C*. *cayetanensis*	*E*. *bieneusi*	*B*. *hominis*	p-value*
CD4 (cells/μL)	266.1 ± 201.3	52.1 ± 50.9^**†**^	153.1 ± 215.8^**†**^	222.3 ± 252.5	303.7 ± 235.5	157.4 ± 146.6^**†**^	0.000
WBC (cells ×10^9^/L)	5.6 ± 2.5	4.4 ± 1.7	6.5 ± 5.1	3.2 ± 1.8^**†**^	7.3 ± 3.0^**†**^	7.4 ± 3.5^**†**^	0.001
NEUT (cells ×10^9^/L)	3.3 ± 2.1	2.7 ± 1.8	4.4 ± 4.7	1.6 ± 0.8^**†**^	4.6 ± 2.6^**†**^	5.4 ± 3.1^**†**^	0.000
LYMPH (cells ×10^9^/L)	1.6 ± 0.8	1.2 ± 0.6	1.4 ± 0.8	1.2 ± 1.1	2.0 ± 0.9^**†**^	1.3 ± 0.7	0.012
MONO (cells ×10^9^/L)	0.4 ± 0.2	0.3 ± 0.1	0.5 ± 0.3	0.4 ± 0.2	0.5 ± 0.2^**†**^	0.5 ± 0.2	0.102
EOSO (cells ×10^9^/L)	0.1 ± 0.2	0.1 ± 0.1	0.1 ± 0.1	0.1 ± 0.02	0.2 ± 0.2^**†**^	0.2 ± 0.4^**†**^	0.291
BASO (cells ×10^9^/L)	0.01 ± 0.02	0.01 ± 0.01	0.02 ± 0.02^**†**^	0.01 ± 0.01	0.01 ± 0.02	0.02 ± 0.06	0.095
RBC (cells ×10^12^/L)	4.6 ± 0.8	4.4 ± 0.7	4.3 ± 0.9	3.2 ± 1.8^**†**^	4.7 ± 0.7	4.4 ± 0.7	0.054
HGB (g/L)	138.8 ± 23.5	129.9 ± 27.0	130.3 ± 25.4	96.5 ± 55.8^**†**^	142.6 ± 19.4	132.6 ± 18.8	0.066

*p-values indicated the comparison among different infection conditions using Kruskal-Wallis test.

^**†**^Statistically different compared with non-infection, using Kruskal-Wallis test.

**Fig 4:**
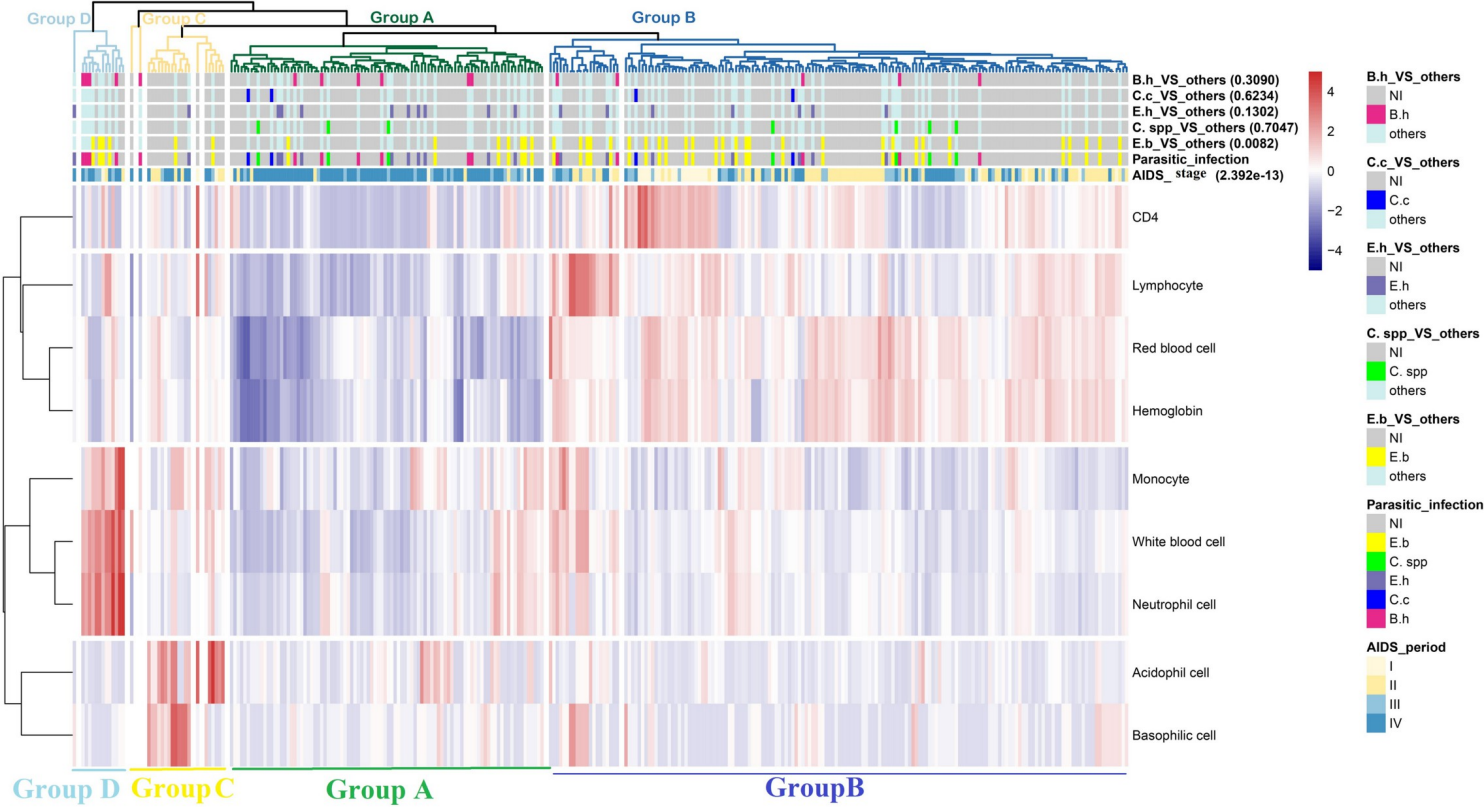
Clustering heatmap of the infection data. Among the 7 color bars, the bottom two are parasitic infection statuses and AIDS periods, and different colors represent different kinds of parasitic infections or different AIDS stages. The remaining 5 color bars are derived from the parasitic infection variable. For instance, in the group of “E.b._VS_other”, samples with parasitic infection of E.b. make up the group of interest, and parts 2 to 5 are separated into another group, while the uninfected group is comprised of samples with no parasitic infection. The other group and the non-infected group are labeled with cyan and gray colors, respectively. The group of interest is marked with a distinct color corresponding to the color in the parasitic infection status (NI, not infected; E.b., *E*. *bieneusi*; C. spp., *Cryptosporidium* spp.; E.h., *E*. *histolytica*; C.c., *C*. *cayetanensis*; B.h., *B*. *hominis*).

CD4^+^ T cell, LYMPH, RBC, and HGB were clustered in a separate branch showing similar patterns in the heat map (Fig 4). WBC, MONO, and NEUT were clustered together, while EOSO and BASO were clustered in another branch. A total of 302 MSM HIV/AIDS individuals that were singly infected with a parasite were divided into four groups. Individuals in groups A and B, accounting for 90% of all individuals, displayed their own unique hemogram patterns.

*E*. *bieneusi*-infected patients were more numerous in group B (*p* = 0.008) (S12 Table), with higher CD4^+^ T cell, LYMPH, RBC, and HGB counts and lower MONO, WBC, NEUT, EOSO, and BASO counts. However, individuals infected with the four other parasites could not be distinguished by these hemogram parameters. We also found that individuals at AIDS stages III/IV were the most numerous in group A (*p* < 0.001), with lower cell counts in the hemogram parameters. In contrast, individuals in early AIDS stages (I/II) were dominant in group B, consistent with the association of *E*. *bieneusi* and the AIDS stage above.

### Species, genotypes/subtypes of enteric parasites

There were three species of *Cryptosporidium*, including *C*. *hominis* (1.8%), *C*. *meleagridis* (1.3%), and *C*. *cuniculus* (0.3%) (Table 6). *C*. *hominis* and *C*. *meleagridis* were the dominant species accounting for 92.3% of *Cryptosporidium* isolates from all HIV/AIDS individuals. In addition, we found three novel subtypes of *C*. *meleagridis*, IIIeA15G2R1 (GenBank accession number MG917667), IIIbA23G1R1d (GenBank accession number MG917668), and IIIgA26G1R1 (GenBank accession number MG917669). This is the first time *C*. *cuniculus* infection is reported in HIV-infected individuals.

**Table 6:** The species, genotypes, and subtypes of *Cryptosporidium*, *E*. *bieneusi*, and *B*. *hominis* in HIV-positive participants.

Routes of HIV infection (n)	*Cryptosporidium*			*E*. *bieneusi*		*B*. *hominis*	
	n (%)	Species (n, %)	Subtype (n)	n (%)	Genotype (n)	n (%)	ST subtype (n)
MSM (308)	9 (2.9)	*C*. *hominis* (6, 1.9)	IbA20G2 (5); NA (1)	45 (14.6)	D (33); EbpC (1); CHN-H1 (4); CHN-H2 (1); CHN-H3 (1); NA (5)	19 (6.2)	ST1 (3); ST3 (15); ST14 (1)
		*C*. *meleagridis* (2, 0.6)	IIIbA23G1R1 (1); NA (1)				
		*C*. *culuculus* (1, 0.3)	NA (1)				
MSW & Others (76)	4 (5.3)	*C*. *hominis* (1, 1.3)	Ia18R4 (1)	11 (14.5)	D (4); NA (7)	12 (15.8)	ST3 (12)
		*C*. *meleagridis* (3, 3.9)	IIIeA15G2R1 (1); IIIgA26G1R1 (2)				

MSM = men having sex with men. MSW = men having sex with women. Others = sexually transmitted women, non-sexually transmitted individuals, and unknown transmission routes were included. NA = not available.

Five genotypes of *E*. *bieneusi* were found among all the HIV/AIDS patients studied (Table 6), including two known genotypes (D and EbpC) and three novel genotypes (CHN-H1, CHN2, and CHN-H3). Genotype D was dominant, being present in 84.1% of all isolates. Genotypes CHN-H1 (GenBank accession number MG255733) and CHN-H2 (GenBank accession number MG255734) showed 98.8% and 98.4% homology with genotype KIN-1 (GenBank accession number KR815514), respectively. Indeed, the genotype CHN-H3 (GenBank accession number MG255735) showed 97.9% homology with genotype EbpC (GenBank accession number KX905207). Phylogenetic analysis indicated that all five genotypes belonged to zoonotic *E*. *bieneusi* group 1 (S1 Fig).

Three ST subtypes of *Blastocystis* were found, including ST1 (9.7%; 3/31), ST3 (87.1%; 27/31), and ST14 (3.2%; 1/31) (Table 6). This is the first time that subtype ST14 is reported in humans.

We found some unique features in MSM HIV/AIDS individuals. For instance, these individuals were infected with a wider range of species or genotypes of *Cryptosporidium*, *E*. *bieneusi*, and *B*. *hominis* than non-MSM subjects. The MSM group had more *C*. *hominis*-infected individuals, and the newly identified *C*. *culuculus* was also found in this group. MSM HIV/AIDS individuals had five genotypes of *E*. *bieneusi*, while the non-MSM group had only one genotype (D). ST1 and ST14 of *B*. *hominis* were only found in MSM individuals (Table 6).

## Discussion

To the best of our knowledge, this study is the first systematic investigation of the epidemiology of five enteric parasites, including *E*. *bieneusi*, *B*. *hominis*, *E*. *histolytica*, *Cryptosporidium* spp., and *C*. *cayetanensis* in HIV/AIDS and MSM HIV/AIDS populations in China. The prevalence of five parasitic infections in HIV/AIDS and MSM HIV/AIDS populations was much higher than in healthy individuals. Considering the lack of information about such infections in the MSM HIV/AIDS population, we could not compare data from the present study with other areas of China. Moreover, to date, only two publications have reported on the epidemic of enteric parasites using molecular tools and indicated that the prevalence of *E*. *bieneusi* was 5.1% in Henan Province and 11.6% in Guangxi Province [16, 17], while that of *Cryptosporidium* spp. was 3.5% in Henan [17]. The prevalence of *E*. *bieneusi* in Heilongjiang Province is higher than that in Guangxi and Henan Province, while that of *Cryptosporidium* spp. is similar to that in Henan. Some reports also revealed various infection rates of *B*. *hominis*, *E*. *histolytica*, and *Cryptosporidium* spp. using morphology or serology methods [5, 14, 15]. It has been well established that these methods may not be adequate to reflect the true epidemic [33].

Epidemiological data suggest that water quality, personal hygiene, animal contact, educational status, ambient temperature, and season are environmental factors associated with parasitic infections [34,35]. However, these factors significantly vary between geographical regions and within different populations [36]. Multivariate logistic regression showed that the warm season was the only statistically significant risk factor for parasitic infection. This risk factor is almost applicable to all parasitic infections except *Cryptosporidium* spp., which might be mainly related to immunodeficiency conditions. Therefore, clinicians and AIDS patients should be aware of the risk of parasitic infections during this season.

In AIDS patients, the prevalence of sexually transmitted infections (STIs) and the severity of other infections are associated with CD4^+^ T cell counts [10]. However, to date, only a limited number of parasites, such as *Cryptosporidium* spp., *E*. *bieneusi*, and *E*. *histolytica*, have been defined as AIDS-associated, and such infections are correlated with low CD4^+^ T cell counts [36–38]. Contradictory findings with *E*. *histolytica* and *E*. *bieneusi* infection were also reported [39, 40]. We found that the low CD4^+^ T cell counts significantly correlated with infection with *Cryptosporidium* spp., *E*. *histolytica*, and *B*. *hominis* but not with *E*. *bieneusi* in HIV/AIDS and MSM HIV/AIDS populations. Therefore, we suggest adding *E*. *histolytica* and *B*. *hominis* as risk parasites for opportunistic infections in AIDS patients and advise examining these infections.

In addition, we also analyzed the haematologic elements of MSM HIV/AIDS individuals and their correlation to parasitic infection. These hemogram parameters can be divided into three branches; LYMPH, RBC, and HGB were clustered in the same branch with CD4^+^ T cell count. These four parameters showed higher counts in the early stages and lower counts in the late stages of AIDS, while the other parameters were generally lower in MSM HIV/AIDS patients. Thus, we suggest that LYMPH, RBC, and HGB can predict the immune status of HIV/AIDS patients together with CD4^+^ T cell count. Parinitha et al. also found that the haematologic manifestations of HIV infection are common and more frequent with disease progression and that there is a significant increase in the number of cases of anemia and lymphopenia with decreasing CD4^+^ T cell counts [41]. This may highlight the importance of simultaneously treating AIDS patients for haematologic manifestations to reduce morbidity. *E*. *bieneusi* and the other four parasites could well be distinguished by CD4^+^ T cell, LYMPH, RBC, and HGB counts. Meanwhile, the severity of AIDS does not increase the infection of *E*. *bieneusi*, the tendency of which is quite different from the four other parasites. We do not know the mechanism of this phenomenon, although there is a similar report [17]. Thus, we need to pay more attention to the high prevalence of *E*. *bieneusi* among AIDS populations and ought to examine HIV-infected individuals with diarrhea even if they are in the early stages of AIDS.

Of the five parasites, *E*. *histolytica*, *Cryptosporidium* spp., *E*. *bieneusi*, and *C*. *cayetanensis* have been found to cause diarrhea associated with HIV, particularly in the context of very low CD4^+^ T cell counts. The influence of *B*. *hominis* infection is still debated [20]. However, when we tried to correlate parasitic infections and diarrhea, we had to consider parasitic infection itself and minimize the influence of non-parasite factors as much as possible. We examined and excluded bacterial co-infection in this study and evaluated the correlation between diarrhea and parasitic infection. The fact that the proportion of PD in the MSM HIV/AIDS group was higher than in the MSW group suggests that MSM may produce a more severe diarrhea situation. Furthermore, the infection proportions of both *Cryptosporidium* spp. and *E*. *histolytica* are slightly higher in PD individuals than in HD individuals. The facts above imply that *E*. *histolytica* or *Cryptosporidium* spp. infection is related to more severe diarrhea in MSM HIV/AIDS individuals. In addition, co-infection of *Cryptosporidium* spp. and *Yersinia enterocolitica* resulted in more severe diarrhea. Furthermore, *E*. *bieneusi* infection was also diarrhea-related but was more significantly associated with HD (moderate cases). *B*. *hominis* was not found to be a diarrhea-related parasite, consistent with other findings [42].

The application of molecular methods for identification and characterization has led to more reliable results, revealed the true prevalence of infections, improved source tracking, calculated host range, and correlated the pathogenic potential of many parasite types [33]. We also analyzed genotypes and the phylogeny of parasites and attempted to track possible sources for high-risk infection routes in HIV/AIDS individuals. In HIV/AIDS individuals, *C*. *hominis* was dominant and possibly correlated with latent infection in local human beings, as it is a species with the human-to-human transmission, and no infection was found in local horses (the sensitive animal) [43, 44]. *C*. *cuniculus* may be derived from local rabbits, while novel subtypes of *C*. *meleagridis* are from animals or humans (S2 Table and S2 Fig). Genotype EbpC was dominant in local children, and D was found in tumor patients [45, 46]. Indeed, genotype D is dominant in various farm animals, while EbpC is dominant in household animals. Therefore, the sources of these two genotypes vary and may be from humans or animals (S1 Table and S1 Fig). Three ST subtypes of *B*. *hominis* were found in HIV/AIDS individuals. ST1 and ST3 are common in humans worldwide. Meanwhile, ST1 and ST3 have been found in local sheep and cattle, and ST3 has been identified in local tumor patients [46, 47]. ST14 has never been identified in humans before. Thus, local cattle and sheep may act as a source of infection for the newly identified ST14, while ST1 and ST3 may be derived from humans, sheep, or cattle (S3 Table). The above findings not only extend the range of zoonotic enteric parasites in humans but also imply the apparent underestimated latent infections by those pathogens and the important source of animals. Extensive investigation of zoonotic enteric parasitic infections, source tracking, and proper prevention strategies should be considered to reduce AIDS-related morbidity and mortality.

Regarding each parasite, we may claim that: 1) *E*. *bieneusi* is highly prevalent in the HIV-infected population and infected individuals with low CD4^+^ T cell counts and diarrhea, especially mild diarrhea, distinguished from the four other parasites. 2) *E*. *histolytica* is related to low CD4^+^ T cell counts and diarrhea, especially severe diarrhea, and is more prevalent at the late stages of AIDS. 3) *Cryptosporidium* spp., like *E*. *histolytica*, are more closely associated with low CD4^+^ T cell counts. 4) *B*. *hominis* infection is associated with low CD4^+^ T cell counts and is more prevalent during the late stages of AIDS but does not occur with diarrhea. Unexpectedly, the positive rate of *B*. *hominis* in MSM was significantly lower than in the MSW population. It is likely that the lower proportion is associated with a high proportion of MSM among the whole HIV/AIDS population because the proportion in females was very high in the present study (26.7%) (S9 Table;), as has been seen elsewhere [48].

This study had some limitations that should be mentioned. 1) There was a significant difference between the number of males and females recruited for the study. 2) The study collected only one stool specimen instead of multiple samples, which could improve the detection rates of different parasites. 3) HIV-negative MSM individuals were not recruited in this study. Although we do not know the prevalence of parasitic infections in the whole MSM population, the main purpose of improving knowledge of co-infection in MSM HIV/AIDS individuals has already been achieved. 4) Most of the enrolled participants had just started ART treatment. Thus, this factor was not taken into the analysis of the influence on parasitic infection. We will conduct continuous follow-ups of these patients to determine the relationship between ART and parasitic infections in the future. 5) The present study was confined to research on MSM HIV/AIDS with enteric pathogens in cold areas; the situation in the tropics may be different. Our study does not represent the entire MSM HIV/AIDS population worldwide.

Currently, studies that reflect actual human infections with enteric parasites are still insufficient. The findings of this study have important implications for public health, the control of AIDS progression, and the control of parasitic infections. First, the presence of asymptomatic carriers of the parasites, the high burden among local animals, and the prevalence in AIDS patients emphasize their importance in the transmission cycle. Therefore, latent infection with enteric parasites in humans and the burden in local animals should be extensively investigated and emphasized to public health workers and patients to prevent and control these infections and decrease mortality in immunosuppressed individuals. Second, source tracking by molecular tools of human infections can more clearly identify possible sources. This is beneficial for adequately managing the local stock breeding industry and blocking transmission routes. The identification of new types or subtypes in humans suggests the evolution of enteric intracellular parasites and a new threat to humans and animals. Third, parasitic infections and diarrhea correlated by eliminating the influence of bacterial pathogens, providing clearer clinical significance for these parasitic infections in the HIV/AIDS population.

In conclusion, there is a common occurrence of the co-infection of enteric intracellular parasites with HIV among MSM from northeast China, and a higher infection proportion was shown in the warm season. The infection of *E*. *histolytica* and *Cryptosporidium* spp. is likely related to more severe diarrhea, while that of *E*. *bieneusi* is related to mild diarrhea. The MSM HIV/AIDS population has a wider range of species or genotypes of *Cryptosporidium*, *E*. *bieneusi*, and *B*. *hominis*. In addition, the possible sources of parasitic infections in these individuals are either humans or animals. Therefore, AIDS patients should be educated and informed to minimize the risk of transmission of parasites from humans and animals.

## Supporting information

S1 Table Prevalence and distribution of zoonotic *E*. *bieneusi* genotypes in different hosts in Heilongjiang Province, China. (DOCX)

S2 Table Prevalence and distribution of zoonotic *Cryptosporidium* species/genotypes and subtypes in different hosts in Heilongjiang Province, China. (DOCX)

S3 Table Prevalence and distribution of Zoonotic *Blastocystis* subtypes (ST) in different hosts in Heilongjiang Province, China. (DOCX)

S4 Table The primers and conditions of the PCR experiment used to identify the parasites. (DOCX)

S5 Table The primers and conditions of the PCR experiment used to identify genotypes of *E*. *bieneusi* and subtypes of *Cryptosporidium*. (DOCX)

S6 Table Sociodemographic, environmental, and clinical profiles of the HIV-positive participants. (DOCX)

S7 Table Sociodemographic, environmental, and clinical profiles of the MSM HIV-positive participants. (DOCX)

S8 Table The participants with mixed infection of parasites. (DOCX)

S9 Table Risk factors associated with enteric parasitic infections in all the HIV-positive participants. (DOCX)

S10 Table Parasitic infection proportions among all HIV-positive participants with different diarrhea statuses. (DOCX)

S11 Table The association of enteric parasitic infections with CD4+ T cell counts of MSM HIV-positive participants. (DOCX)

S12 Table The association of parasite infection with the cluster groups. (DOCX)

S1 Fig Phylogenetic relationship of *E*. *bieneusi* genotypes identified in the present study and known genotypes found in Heilongjiang Province, China, deposited in GenBank. (DOCX)

S2 Fig Phylogenetic relationship of gp60 subtypes of *C*. *meleagridis*. (DOCX)
