# Supplementary material for: A baseline epidemiological study of the co-infection of enteric protozoans with human immunodeficiency virus among men who have sex with men from Northeast China
Source: PLoS Negl Trop Dis. 2022 Sep 6;16(9):e0010712. doi: 10.1371/journal.pntd.0010712 (PMC9447920; doi:10.1371/journal.pntd.0010712)
Supplement: S1 Table — (DOCX) [file pntd.0010712.s001.docx]

**S1Table Prevalence and distribution of zoonotic *E. bieneusi* genotypes in different hosts in Heilongjiang Province, China**

| **Host** | **n** | **Positive**  **n (%)** | **Zoonotic genotypes (%)** | | **References** |
| --- | --- | --- | --- | --- | --- |
|  |  |  | **Group1** | **Group2** |  |
| Farmed | | | | | |
| Pig | 95 | 85 (89.5) | EbpA (31.6); D (20.0); H (18.9); O (11.6) | Not found | [1] |
| Cattle | 139 | 43 (30.1) | O (18.7); EbpA (1.4); D (0.7) | I (1.4); J (1.4); BEB4 (0.7) | [2] |
|  | 537 | 32 (6.0) | CS-4 (1.1); EbpC (0.6) | J (2.0); BEB4 (0.37); I (0.6) | [3] |
| Fox | 110 | 18 (16.4) | D (11.8); EbpC (4.5) | Not found | [4] |
|  | 191 | 53 (27.7) | D (23.0) | Not found | [5] |
| Rex rabbits | 150 | 22 (14.7) | D (2.0); Type IV (1.3); Peru6 (0.7) | I (0.7) | [6] |
| Raccoon dogs | 40 | 1 (2.5) | D(2.5) | Not found | [7] |
|  | 162 | 17 (10.5) | D (8.6) | Not found | [5] |
|  | 49 | 1 (4.1) | D (2.0) | Not found | [4] |
| Reindeer | 145 | 21 (16.8) | Peru6 ( 4.1) | Not found | [8] |
| Sika deer | 86 | 29 (32.6) | - | BEB6 ( 23.3) | [9] |
| Subtotal | 1704 | 322 (18.9) | D (5.6); O (2.2); EbpA (1.9); H (1.1); EbpC (0.5); Peru6 (0.4); CS-4 (0.4); Type IV (0.1) | BEB6 ( 1.2); J (0.8); I(0.4); BEB4 (0.2) |  |
| House-hold | | | | | |
| Cat | 52 | 3 (1.9) | D(3.8); Type IV(1.9) | Not found | [10] |
| Chicken | 11 | 2 (18.2) | Henan-IV (18.2) | Not found | [11] |
| Cranes | 56 | 7 (12.5) | D (5.4); Peru6 (3.6); EbpA (1.8) | Not found | [12] |
| Duck | 62 | 6 (9.7) | Peru6 (3.2) | BEB6 (1.6) | [12] |

**S1 Table Prevalence and distribution of zoonotic *E. bieneusi* genotypes in different hosts in Heilongjiang Province, China (Continued)**

| **Host** | **n** | **Positive**  **n (%)** | **Zoonotic genotypes (%)** | | **References** |
| --- | --- | --- | --- | --- | --- |
|  |  |  | **Group1** | **Group2** |  |
| Dog | 267 | 18 (6.7) | EbpC (0.7); D (0.4) | Not found | [10] |
| Goat | 55 | 12 (21.8) | Peru6 (5.5); D (3.6); EbpC (3.6); EbpA (1.8) | BEB6 (5.5) | [13] |
| Goose | 26 | 8 (30.8) | Peru6 (11.5) | BEB6 (15.4) | [12] |
| Pig | 49 | 3 (6.1) | EbpC (6.1) | Not found | [11] |
|  | 86 | 39 (45.3) | EbpC (11.6); Henan-IV(4.7); O(3.5); EbpA(3.5); CS-4 (2.3); D (1.2); EbpD (1.2) | Not found | [14] |
| Pigeon | 50 | 22 (44.0) | Peru6 (44.0) | Not found | [12] |
| Sheep | 138 | 31 (22.5) | Peru6 (3.6); D (2.9); O (2.2) | BEB6 (8.7) | [13] |
|  | 45 | 2 (4.4) | - | BEB6 (4.4) | [15] |
|  | 489 | 68 (13.9) | CS-4 (1.0); EbpC (0.2) | BEB6 (8.6) | [3] |
| Subtotal | 1386 | 221 (15.9) | Peru6 (2.7); EbpC (1.3); D (1.3); CS-4 (0.5); Henan-IV (0.4); O (0.4); EbpA (0.4); TypeIV (0.1); EbpD (0.1) | BEB6 (4.6) |  |
| Human | | | | | |
| Healthy | 199 | 2(1.0) | D (1.0) | Not found | [16] |
| Cancer | 381 | 5(1.3) | D (1.0); HLJCP-1 (0.3) | Not found | [17] |
| HIV | 384 | 56 (14.6) | D (37); EbpC (1); CHN-H1 (4); CHN-H2(1); CHN-H3 (1); CHN-H4 (1) | Not found | This study |

References

1. Zhao W, Zhang W, Yang F et al. High prevalence of *Enterocytozoon bieneusi* in asymptomatic pigs and assessment of zoonotic risk at the genotype level. Appl Environ Microbiol. 2014; 80: 3699-3707.
2. Zhao W, Zhang W, Yang F, et al. *Enterocytozoon bieneusi* in Dairy Cattle in the Northeast of China: Genetic Diversity of ITS Gene and Evaluation of Zoonotic Transmission Potential. J Eukaryot Microbiol. 2015; 62: 553-60.
3. Jiang Y, Tao W, Wan Q, et al. Zoonotic and Potentially Host-Adapted *Enterocytozoon bieneusi* Genotypes in Sheep and Cattle in Northeast China and an Increasing Concern about the Zoonotic Importance of Previously Considered Ruminant-Adapted Genotypes. Appl Environ Microbiol. 2015; 81: 3326-35.
4. Zhao W, Zhang W, Yang Z, et al. Genotyping of *Enterocytozoon bieneusi* in Farmed Blue Foxes (*Alopex lagopus*) and Raccoon Dogs (*Nyctereutes procyonoides*) in China. PLoS One. 2015; 10: e0142611.
5. Yang Y, Lin Y, Li Q, et al. Widespread presence of human-pathogenic *Enterocytozoon bieneusi* genotype D in farmed foxes (*Vulpes vulpes*) and raccoon dogs (*Nyctereutes procyonoides*) in China: first identification and zoonotic concern. Parasitol Res. 2015; 114: 4341-8.
6. Yang Z, Zhao W, Shen Y, et al. Subtyping of *Cryptosporidium cuniculus* and genotyping of *Enterocytozoon bieneusi* in rabbits in two farms in Heilongjiang Province, China. Parasite. 2016; 23: 52.
7. Xu C, Ma X, Zhang H, et al. Prevalence, risk factors and molecular characterization of *Enterocytozoon bieneusi* in raccoon dogs (*Nyctereutes procyonoides*) in five provinces of Northern China. Acta Trop. 2016; 161: 68-72.
8. Liu W, Nie C, Zhang L, et al. First detection and genotyping of *Enterocytozoon bieneusi* in reindeers (*Rangifer tarandus*): a zoonotic potential of ITS genotypes. Parasit Vectors. 2015; 8: 526.
9. Zhao W, Zhang W, Wang R, et al. *Enterocytozoon bieneusi* in sika deer (*Cervus nippon*) and red deer (*Cervus elaphus*): deer specificity and zoonotic potential of ITS genotypes. Parasitol Res. 2014; 113: 4243-50.
10. Li W, Li Y, Song M, et al. Prevalence and genetic characteristics of *Cryptosporidium*, *Enterocytozoon bieneusi* and *Giardia duodenalis* in cats and dogs in Heilongjiang province, China. Vet Parasitol. 2015; 208: 125-34.
11. Li W, Tao W, Jiang Y, et al. Genotypic distribution and phylogenetic characterization of *Enterocytozoon bieneusi* in diarrheic chickens and pigs in multiple cities, China: potential zoonotic transmission. PLoS One. 2014; 9: e108279.
12. Zhao W, Yu S, Yang Z, et al. Genotyping of *Enterocytozoon bieneusi* (*Microsporidia*) isolated from various birds in China. Infect Genet Evol. 2016; 40: 151-4.
13. Zhao W, Zhang W, Yang D, et al. Prevalence of *Enterocytozoon bieneusi* and genetic diversity of ITS genotypes in sheep and goats in China. Infect Genet Evol. 2015; 32: 265-70.
14. Wan Q, Lin Y, Mao Y, et al. High Prevalence and Widespread Distribution of Zoonotic *Enterocytozoon bieneusi* Genotypes in Swine in Northeast China: Implications for Public Health. J Eukaryot Microbiol. 2016; 63: 162-70.
15. Li W, Li Y, Li W, et al. Genotypes of *Enterocytozoon bieneusi* in livestock in China: high prevalence and zoonotic potential. PLoS One. 2014; 9: e97623.
16. Yang J, Song M, Wan Q, et al. *Enterocytozoon bieneusi* genotypes in children in Northeast China and assessment of risk of zoonotic transmission. J Clin Microbiol. 2014; 52: 4363-7.
17. Zhang W, Ren G, Zhao W, et al. Genotyping of *Enterocytozoon bieneusi* and Subtyping of *Blastocystis* in Cancer Patients: Relationship to Diarrhea and Assessment of Zoonotic Transmission. Front Microbiol. 2017; 8: 1835.
