# Supplementary material for: A baseline epidemiological study of the co-infection of enteric protozoans with human immunodeficiency virus among men who have sex with men from Northeast China
Source: PLoS Negl Trop Dis. 2022 Sep 6;16(9):e0010712. doi: 10.1371/journal.pntd.0010712 (PMC9447920; doi:10.1371/journal.pntd.0010712)
Supplement: S2 Table — (DOCX) [file pntd.0010712.s002.docx]

**S2 Table Prevalence and distribution of zoonotic *Cryptosporidium* species/genotypes and subtypes in animal hosts in Heilongjiang Province, China**

| **Host** | **n** | **Positive n (%)** | **Zoonotic *Cryptosporidium* (%)** | | **References** |
| --- | --- | --- | --- | --- | --- |
|  |  |  | **Species/genotypes** | **Subtypes** |  |
| Cat | 52 | 2 (3.8) | *C. felis*(1.9); *C. parvum* (1.9) | Not applicable or not examined | 1 |
| Cattle | 507 | 27 (5.3) | *C. andersoni* (5.1) | Not examined | 2 |
|  | 151 | 72 (47.68) | *C. andersoni* (17.2) | Not examined | 3 |
|  |  |  | *C. meleagridis* (3.3) | IIIeA22G2R1(2.6) |  |
|  |  |  | *C. parvum* (1.3) | IIdA19G1(1.3) |  |
|  | 537 | 130 (24.2) | *C. parvum* (16.2) | IIdA15G1 (4.5); IIdA20G1 (8.9) | 4 |
|  |  |  | *C. andersoni* (0.4); *Suis*-like genotype (0.4) | Not applicable or not examined |  |
| Dog | 267 | 6 (2.2) | *C. canis* (1.9); *C. ubiquitum* (0.4) | Not applicable or not examined | 1 |
| Foxes | 70 | 16 (22.9) | *C. canis* (22.9) | Not applicable | 5 |
|  | 191 | 3 (1.6) | *C. canis* (1.6) | Not applicable | 6 |
|  | 107 | 11 (10.3) | *C. canis* (10.3) | Not applicable | 7 |
| Horse | 29 | 2 (6.9) | *C. andersoni* (6.9) | A4, A4, A4, A1 (6.9) | 8 |
| Minks | 162 | 48 (29.6) | *C. canis* (11.7); *C. meleagridis* (1.9) | Not applicable or not examined | 7 |
|  |  |  | mink genotype (11.1) | Xb, Xc, Xd |  |
| Pet-derived | 151 | 14 (9.3) | *C. ubiquitum* (9.3) | Not examined | 9 |
| Pig | 113 | 63 (55.8) | *C. scrofarumn* (28.3); *C. suis* (27.4) | Not applicable | 10 |
| Rabbit | 65 | 19 (29.2) | *C. cuniculus* (29.2) | VbA28 (3.1); VbA29 (24.6) | 11 |
|  | 378 | 9 (2.38) | *C. cuniculus* (2.4) | VbA32( 0.8); VbA21 ( 1.6) | 12 |
| Raccoon dogs | 162 | 17 (10.5) | *C. canis* (8.0) | Not applicable | 7 |
| Rex rabbits | 150 | 5 (3.3) | *C. cuniculus* (3.3) | VbA32 (3.3) | 11 |
| HIV | 384 | 13 (3.4) | *C.hominis* (7); *C.meleagridis* (5); *C.cuniculus* (1) | Ia18R4(1);IbA20G2 (6);IIIeA15G2R1;IIIbA23G1R1;IIIgA26G1R1 | This study |

References

1. Li W, Li Y, Song M, et al. Prevalence and genetic characteristics of *Cryptosporidium*, *Enterocytozoon bieneusi* and *Giardia duodenalis* in cats and dogs in Heilongjiang province, China. Vet Parasitol. 2015; 208: 125-34.
2. Liu A, Wang R, Li Y, et al. Prevalence and distribution of *Cryptosporidium* spp. in dairy cattle in Heilongjiang Province, China. Parasitol Res. 2009; 105: 797-802.
3. Zhang W, Wang R, Yang F, et al. Distribution and genetic characterizations of *Cryptosporidium* spp. in pre-weaned dairy calves in Northeastern China's Heilongjiang Province. PLoS One. 2013; 8: e54857.
4. Tao W, Li Y, Yang H, et al. Widespread Occurrence of Zoonotic *Cryptosporidium* Species and Subtypes in Dairy Cattle from Northeast China: Public Health Concerns. J Parasitol. 2017.
5. Zhang XX, Cong W, Ma JG, et al. First report of *Cryptosporidium canis* in farmed Arctic foxes (*Vulpes lagopus*) in China. Parasit Vectors. 2016; 9: 126.
6. Zhang S, Tao W, Liu C, et al. First report of *Cryptosporidium canis* in foxes (*Vulpes vulpes*) and raccoon dogs (*Nyctereutes procyonoides*) and identification of several novel subtype families for *Cryptosporidium mink* genotype in minks (*Mustela vison*) in China. Infect Genet Evol. 2016; 41: 21-25.
7. Yang Z, Zhao W, Wang J, Ren G, Zhang W, Liu A. Molecular detection and genetic characterizations of *Cryptosporidium* spp. in farmed foxes, minks, and raccoon dogs in northeastern China. Parasitol Res. 2018; 117: 169-175.
8. Liu A, Zhang J, Zhao J, et al. The first report of *Cryptosporidium andersoni* in horses with diarrhea and multilocus subtype analysis. Parasit Vectors. 2015; 8: 483.
9. Li Q, Li L, Tao W, et al. Molecular investigation of *Cryptosporidium* in small caged pets in northeast China: host specificity and zoonotic implications. Parasitol Res. 2016; 115: 2905-11.
10. Zhang W, Yang F, Liu A, et al. Prevalence and genetic characterizations of *Cryptosporidium* spp. in pre-weaned and post-weaned piglets in Heilongjiang Province, China. PLoS One. 2013; 8: e67564.
11. Yang Z, Zhao W, Shen Y, et al. Subtyping of *Cryptosporidium cuniculus* and genotyping of *Enterocytozoon bieneusi* in rabbits in two farms in Heilongjiang Province, China. Parasite. 2016; 23: 52.
12. Zhang W, Shen Y, Wang R, et al. *Cryptosporidium cuniculus* and *Giardia duodenalis* in rabbits: genetic diversity and possible zoonotic transmission. PLoS One. 2012; 7: e31262.
