# Supplementary material for: A baseline epidemiological study of the co-infection of enteric protozoans with human immunodeficiency virus among men who have sex with men from Northeast China
Source: PLoS Negl Trop Dis. 2022 Sep 6;16(9):e0010712. doi: 10.1371/journal.pntd.0010712 (PMC9447920; doi:10.1371/journal.pntd.0010712)
Supplement: S3 Table — (DOCX) [file pntd.0010712.s003.docx]

**S3 Table Prevalence and distribution of Zoonotic *Blastocystis* subtypes (ST) in different hosts in Heilongjiang Province, China**

| **Host** | **n** | | **Positive number (%)** | **Zoonotic STs (%)** | **References** |
| --- | --- | --- | --- | --- | --- |
| Cattle | | 526 | 54 (10.3) | ST14 (1.9), ST4 (0.4), ST5 (0.2) | 1 |
| Cattle | | 147 | 14 (9.5) | ST3 (1.4), ST14 (1.4) | 2 |
| Pig | | 68 | 6 (8.8) | ST5 (8.8) | 2 |
| Sheep | | 109 | 6 (5.5) | ST1 (0.9), ST5 (0.9), ST14 (0.9) | 3 |
| Cancer | | 381 | 27 (7.1) | ST 1(3.1), ST3 (3.9) | 3 |
| HIV | | 384 | 31(8.1) | ST1 (3); ST3 (27); ST14 (1) | This study |

1. Zhu W, Tao W, Gong B, et al. First report of *Blastocystis* infections in cattle in China. Vet Parasitol. 2017; 246: 38-42.
2. Wang J, Gong B, Yang F, et al. Subtype distribution and genetic characterizations of *Blastocystis* in pigs, cattle, sheep and goats in northeastern China's Heilongjiang Province. Infect Genet Evol. 2018; 57: 171-176.
3. Zhang W, Ren G, Zhao W, et al. Genotyping of *Enterocytozoon bieneusi* and Subtyping of *Blastocystis* in Cancer Patients: Relationship to Diarrhea and Assessment of Zoonotic Transmission. Front Microbiol. 2017; 8: 1835.
