# Supplementary material for: A baseline epidemiological study of the co-infection of enteric protozoans with human immunodeficiency virus among men who have sex with men from Northeast China
Source: PLoS Negl Trop Dis. 2022 Sep 6;16(9):e0010712. doi: 10.1371/journal.pntd.0010712 (PMC9447920; doi:10.1371/journal.pntd.0010712)
Supplement: S4 Table — (DOCX) [file pntd.0010712.s004.docx]

**S4 Table The primers and target fragment sizes the PCR of the five protozoa.**

| **Protozoa** | Gene locus | Primers (5 '-3') | Annealing temp (°C) | Expected product size (bp) | Ref |
| --- | --- | --- | --- | --- | --- |
| *Cryptosporidium* | SSU rRNA | F1:TTCTAGAGCTAATACATGCG | 55 | 1325 | [1] |
|  |  | R1:CCCATTTCCTTCGAAACAGGA |  |  |  |
|  |  | F2:GGAAGGGTTGTATTTATTAGATAAAG | 55 | 830 |  |
|  |  | R2:AAGGAGTAAGGAACAACCTCCA |  |  |  |
| *E. histolytica* | SSU rRNA | F1:TGCTGTGATTAAAACGCT | 58 | 1076 | [2] |
|  |  | R1:TTAACTATTTCAATCTCGG |  |  |  |
|  |  | F2:ACATTTTGAAGACTTTATGTAAGTA | 57 | 427 |  |
|  |  | R2:CAGATCTAGAAACAATGCTTCTCT |  |  |  |
| *C. cayetanensis* | 18S rRNA | F1:ATGTAAAACCCTTCCAGAGTAAC | 55 | 1000 | [3] |
|  |  | R1:GCAATAATCTATCCCCATCACG |  |  |  |
|  |  | F2:AATTCCAGCTCCAATAGTGTAT | 55 | 500 |  |
|  |  | R2:CAGGAGAAGCCAAGGTAGGCRTTT |  |  |  |
| *E. bieneusi* | SSU rRNA | F1:CACCAGGTTGATTCTGCG | 55 | 1200 | 4] |
|  |  | R1:GTGACGGGCGGTGTGTAC |  |  |  |
|  |  | F2:GAAACTTGTCCACTCCTTACG | 55 | 607 |  |
|  |  | R2:CCATGCACCACTCCTGCCATT |  |  |  |
| *B. hominis* | SSU-rDNA barcode | F：ATCTGGTTGATCCTGCCAGT | 55 | 600 | [5] |
|  |  | R：GAGCTTTTTAACTGCAACAACG |  |  |  |

**References**

1. Xiao L, Escalante L, Yang C, et al. Phylogenetic analysis of *Cryptosporidium* parasites based on the small-subunit rRNA gene locus. Appl Environ Microbiol. 1999; 65: 1578-83.
2. Paglia MG, Visca P. An improved PCR-based method for detection and differentiation of *Entamoeba histolytica* and *Entamoeba dispar* in formalin-fixed stools. Acta Trop. 2004; 92: 273-7.
3. Li G, Xiao S, Zhou R, Li W, Wadeh H. Molecular characterization of *Cyclospora*-like organism from dairy cattle. Parasitol Res. 2007; 100: 955-61.
4. Zhao W, Zhang W, Yang F, et al. High prevalence of *Enterocytozoon bieneusi* in asymptomatic pigs and assessment of zoonotic risk at the genotype level. Appl Environ Microbiol. 2014; 80: 3699-3707.
5. Scicluna SM, Tawari B, Clark CG. DNA barcoding of *blastocystis*. Protist. 2006; 157: 77-85.
