# Supplementary material for: A baseline epidemiological study of the co-infection of enteric protozoans with human immunodeficiency virus among men who have sex with men from Northeast China
Source: PLoS Negl Trop Dis. 2022 Sep 6;16(9):e0010712. doi: 10.1371/journal.pntd.0010712 (PMC9447920; doi:10.1371/journal.pntd.0010712)
Supplement: S5 Table — (DOCX) [file pntd.0010712.s005.docx]

**S5 Table Primers and target fragment sizes for PCR of ITS and Gp60 genes of *E. bieneusi* and *Cryptosporidium*, respectively.**

| **Protozoa** | Gene locus | Primers (5 '-3') | Annealing  temp (°C) | Expected product size (bp) | Ref |
| --- | --- | --- | --- | --- | --- |
| *Cryptosporidium* | gp60 | F1: ATA GTC TCC GCT GTA TTC | 55 | 1200 | [1] |
|  |  | R1: TCC GCT GTA TTC TCA GCC |  |  |  |
|  |  | F2: GGA AGG AAC GAT GTA TCT | 55 | 800 ~ 850 |  |
|  |  | R2: GCA GAG GAA CCA GCA TC |  |  |  |
| *E. bieneusi* | ITS | F1: GGT CAT AGG GAT GAA GAG | 55 | 410 | [2] |
|  |  | R1: TTC GAG TTC TTT CGC GCT C |  |  |  |
|  |  | F2: GCT CTG AAT ATC TAT GGC T | 55 | 389 |  |
|  |  | R2: ATC GCC GAC GGA TCC AAG TG |  |  |  |

**References**

1. Buckholt MA, Lee JH, Tzipori S. Prevalence of *Enterocytozoon bieneusi* in swine: an 18-month survey at a slaughterhouse in Massachusetts. Appl Environ Microbiol. 2002; 68: 2595-9.
2. Alves M, Xiao L, Sulaiman I, Lal AA, Matos O, Antunes F. Subgenotype analysis of *Cryptosporidium* isolates from humans, cattle, and zoo ruminants in Portugal. J Clin Microbiol. 2003; 41: 2744-7.
