# Supplementary material for: A baseline epidemiological study of the co-infection of enteric protozoans with human immunodeficiency virus among men who have sex with men from Northeast China
Source: PLoS Negl Trop Dis. 2022 Sep 6;16(9):e0010712. doi: 10.1371/journal.pntd.0010712 (PMC9447920; doi:10.1371/journal.pntd.0010712)
Supplement: S6 Table — (DOCX) [file pntd.0010712.s006.docx]

**S6 Table Socio-demographic, environmental and clinical profiles of the HIV-positive participants**

| **Characteristics** | | **No. of participants** | **Proportion (%)** |
| --- | --- | --- | --- |
| Gender | Male | 369 | 96.1 |
|  | Female | 15 | 3.9 |
| Age | 17-30 | 134 | 34.9 |
|  | 31-50 | 186 | 48.4 |
|  | >50 | 64 | 16.7 |
| Occupation | Farmer | 128 | 33.3 |
|  | No-farmer | 256 | 66.7 |
| Drinking boiled water | Yes | 256 | 66.7 |
|  | No | 128 | 33.3 |
| Contact with animal | Yes | 123 | 30.0 |
|  | No | 261 | 70.0 |
| Season | Nov.-Apr | 150 | 39.1 |
|  | May.-Oct | 234 | 60.9 |
| ART | Yes | 158 | 41.1 |
|  | No | 226 | 58.9 |
| Antibiotic | Yes | 250 | 65.1 |
|  | No | 134 | 34.9 |
| CD4^+^T | <150 | 155 | 40.4 |
|  | 150-350 | 114 | 29.7 |
|  | >350 | 115 | 29.9 |
| Diarrhea state | Yes | 178 | 46.4 |
|  | No | 206 | 53.6 |
| AIDS stages | I | 41 | 10.7 |
|  | II | 96 | 25.0 |
|  | III | 78 | 20.3 |
|  | IV | 169 | 44.0 |
| VL | <1000 | 108 | 28.1 |
|  | 1000-100000 | 184 | 47.9 |
|  | >100000 | 92 | 24.0 |
| Route of infection with HIV | MSM | 308 | 80.2 |
|  | MSW | 31 | 8.1 |
|  | Other | 45 | 11.7 |
| Total |  | 384 | 100 |

MSM=men who have sex with men. MSW=men who have sex with women. Others=sexually transmitted women, non-sexually transmitted individuals and unknown transmission routs were included. ART =antiretroviral therapy. VL=viral load.
