# Supplementary material for: A baseline epidemiological study of the co-infection of enteric protozoans with human immunodeficiency virus among men who have sex with men from Northeast China
Source: PLoS Negl Trop Dis. 2022 Sep 6;16(9):e0010712. doi: 10.1371/journal.pntd.0010712 (PMC9447920; doi:10.1371/journal.pntd.0010712)
Supplement: S7 Table — (DOCX) [file pntd.0010712.s007.docx]

**S7 Table Socio-demographic, environmental and clinical profiles of the MSM HIV-positive participants**

| **Characteristics** | | **No. of participants** | **Proportion (%)** |
| --- | --- | --- | --- |
| Age | 17-30 | 131 | 42.5 |
|  | 31-50 | 142 | 46.1 |
|  | >50 | 35 | 11.4 |
| Occupation | Farmer | 86 | 27.9 |
|  | No-farmer | 222 | 72.1 |
| Drinking boiled water | Yes | 207 | 67.2 |
|  | No | 101 | 32.8 |
| Contact with animal | Yes | 97 | 31.5 |
|  | No | 211 | 68.5 |
| Season | Nov.-Apr | 111 | 36.0 |
|  | May.-Oct | 197 | 64.0 |
| ART | Yes | 122 | 39.6 |
|  | No | 186 | 60.4 |
| Antibiotic | Yes | 190 | 61.7 |
|  | No | 118 | 38.3 |
| CD4^+^T | <150 | 121 | 39.3 |
|  | 150-350 | 97 | 31.5 |
|  | >350 | 90 | 29.2 |
| Diarrhea state | Yes | 137 | 44.5 |
|  | No | 171 | 55.5 |
| AIDS stages | I | 34 | 11.0 |
|  | II | 84 | 27.3 |
|  | III | 56 | 18.2 |
|  | IV | 134 | 43.5 |
| VL | <1000 | 77 | 25.0 |
|  | 1000-100000 | 152 | 49.4 |
|  | >100000 | 79 | 25.6 |
| Total |  | 308 | 100 |

MSM=men who have sex with men. ART =antiretroviral therapy. VL=viral load.
