# Supplementary material for: A baseline epidemiological study of the co-infection of enteric protozoans with human immunodeficiency virus among men who have sex with men from Northeast China
Source: PLoS Negl Trop Dis. 2022 Sep 6;16(9):e0010712. doi: 10.1371/journal.pntd.0010712 (PMC9447920; doi:10.1371/journal.pntd.0010712)
Supplement: S8 Table — (DOCX) [file pntd.0010712.s008.docx]

**S8 Table The participants with mixed infection of parasites**

| **Participant ID** | **Routes of HIV infection** | **CD4+T cell counts** | **AIDS stages** | **Diarrhea states** | **Parasites** |
| --- | --- | --- | --- | --- | --- |
| CHLJ16087 | MSW | 20 | IV | PD | *C. meleagridis + E. histolytica* |
| CHLJ16088 | MSM | 51 | IV | PD | *C. hominis + E. bieneusi* |
| CHLJ16092 | MSM | 54 | IV | PD | *C. meleagridis + E. histolytica* |
| CHLJ16123 | MSM | 121 | IV | ND | *B. hominis + E. bieneusi* |
| CHLJ16246 | MWS | 453 | II | PD | *B. hominis + E. bieneusi* |
| CHLJ16126 | MSM | 489 | III | ND | *B. hominis + E. bieneusi* |
| CHLJ16249 | MSM | 276 | IV | PD | *B. hominis + E. bieneusi* |
| CHLJ-CRB16038 | Other | 160 | III | PD | *E. histolytica + E. bieneusi* |
| CHLJ16085 | MSW | 70 | IV | HD | *C. meleagridis + E. bieneusi* |
| CHLJ16237 | MSM | 100 | IV | HD | *B. hominis + E. bieneusi* |

MSM=men who have sex with men. MSW=men who have sex with women. Others=sexually transmitted women, non-sexually transmitted individuals and unknown transmission routs were included. PD=present diarrhea. HD=historic diarrhea, as described in the methods. ND=non-diarrhea.
