# Supplementary material for: A baseline epidemiological study of the co-infection of enteric protozoans with human immunodeficiency virus among men who have sex with men from Northeast China
Source: PLoS Negl Trop Dis. 2022 Sep 6;16(9):e0010712. doi: 10.1371/journal.pntd.0010712 (PMC9447920; doi:10.1371/journal.pntd.0010712)
Supplement: S9 Table — (DOCX) [file pntd.0010712.s009.docx]

**S9 Table Risk factors associated with enteric parasite infection among all HIV-positive participants**

| Characteristics | | n | Any of parasites | |  | *E. histolytica* | |  | *E. bieneusi* | |  | *Cryptosporidium* | |  | *C. cayetanensis* | |  | *B. hominis* | |
| --- | --- | --- | --- | --- | --- | --- | --- | --- | --- | --- | --- | --- | --- | --- | --- | --- | --- | --- | --- |
|  |  |  | n (%) | p value |  | n (%) | p value |  | n (%) | p value |  | n (%) | p value |  | n (%) | p value |  | n (%) | p value |
| Gender | |  |  |  |  |  |  |  |  |  |  |  |  |  |  |  |  |  |  |
|  | Male | 369 | 115 (31.2) | 0.661 |  | 24 (6.5) | 0.612* |  | 55 (14.9) | 0.608 |  | 13 (3.5) | 1.000* |  | 6 (1.6) | 0.656 |  | 27 (7.3) | 0.027 |
|  | Female | 15 | 6 (40.0) |  |  | 0 |  |  | 1 (6.7) |  |  | 0 |  |  | 1 (6.7) |  |  | **4 (26.7)** |  |
| Occupation | |  |  |  |  |  |  |  |  |  |  |  |  |  |  |  |  |  |  |
|  | Farmer | 128 | **54 (42.2)** | 0.002 |  | 4 (3.1) | 0.078* |  | **29 (22.7)** | 0.002 |  | 7 (5.5) | 0.195 |  | 2 (1.6) | 1.000* |  | **16 (12.5)** | 0.029 |
|  | Non-farmer | 256 | 67 (26.2) |  |  | 20 (7.8) |  |  | 27 (10.5) |  |  | 6 (2.3) |  |  | 5 (2.0) |  |  | 15 (5.9) |  |
| Drinking boiled water | | |  |  |  |  |  |  |  |  |  |  |  |  |  |  |  |  |  |
|  | Yes | 256 | 75 (29.3) | 0.187 |  | 17 (6.6) | 0.655 |  | 37 (14.5) | 0.919 |  | 5 (2.0) | 0.028 |  | 4 (1.6) | 0.893 |  | 20 (7.8) | 0.791 |
|  | No | 128 | 46 (35.9) |  |  | 7 (5.5) |  |  | 19 (14.8) |  |  | **8 (6.3)** |  |  | 3 (2.3) |  |  | 11 (8.6) |  |
| Contact with animal | | |  |  |  |  |  |  |  |  |  |  |  |  |  |  |  |  |  |
|  | Yes | 123 | 46 (37.4) | 0.088 |  | 10 (8.1) | 0.296 |  | 20 (16.3) | 0.523 |  | 5 (4.1) | 0.613 |  | 1 (0.8) | 0.544 |  | 13 (10.6) | 0.218 |
|  | No | 261 | 75 (28.7) |  |  | 14 (5.4) |  |  | 36 (13.8) |  |  | 8 (3.1) |  |  | 6 (2.3) |  |  | 18 (6.9) |  |
| Season | |  |  |  |  |  |  |  |  |  |  |  |  |  |  |  |  |  |  |
|  | Nov.-Apr. | 234 | 57 (24.4) | 0.000 |  | 7 (3.0) | 0.001 |  | 22 (9.4) | 0.000 |  | 8 (3.4) | 0.964 |  | 2 (0.9) | 0.167 |  | 11 (6.0) | 0.005 |
|  | May.-Oct. | 150 | **64 (42.7)** |  |  | **17 (11.3)** |  |  | **34 (22.7)** |  |  | 5 (3.3) |  |  | 5 (3.3) |  |  | **20 (13.3)** |  |

**S9 Table Risk factors associated with enteric parasite infection among all HIV-positive participants (Continued)**

| Characteristics | | No. | Any of parasites | |  | *E. histolytica* | |  | *E. bieneusi* | |  | *Cryptosporidium* spp. | |  | *C. cayetanensis* | |  | *B. hominis* | |
| --- | --- | --- | --- | --- | --- | --- | --- | --- | --- | --- | --- | --- | --- | --- | --- | --- | --- | --- | --- |
|  |  |  | n (%) | p value |  | n (%) | p value |  | n (%) | p value |  | n (%) | p value |  | n (%) | p value |  | n (%) | p value |
| Route of infection | | |  |  |  |  |  |  |  |  |  |  |  |  |  |  |  |  |  |
|  | MSM^a^ | 308 | 92 (29.9) | 0.290 |  | 19 (6.2) | 0.709 |  | 45 (14.6) | 0.197 |  | 9 (2.9) | 0.599 |  | 5 (1.6) | 0.947 |  | 19 (6.2) | 0.039 |
|  | MSW | 31 | 11 (35.5) |  |  | 3 (9.7) |  |  | 5 (16.1) |  |  | 2 (6.5) |  |  | 1 (3.2) |  |  | **5 (16.1)** |  |
|  | Subtotal^b^ | 339 | 103 (30.4) | 0.493 |  | 22 (6.5) | 0.561 |  | 50 (14.7) | 0.253 |  | 11 (3.2) | 1.000* |  | 6 (1.8) | 1.000* |  | 24 (7.1) | 0.020 |
|  | Other | 45 | 18 (40.0) |  |  | 2 (4.0) |  |  | 6 (13.3) |  |  | 2 (4.0) |  |  | 1 (2.0) |  |  | **7 (15.6)** |  |
| Antibiotic | |  |  |  |  |  |  |  |  |  |  |  |  |  |  |  |  |  |  |
|  | Yes | 250 | 82 (32.8) | 0.457 |  | 17 (6.8) | 0.543 |  | 35 (14.0) | 0.658 |  | 10 (4.0) | 0.363 |  | 4 (1.6) | 0.963 |  | 25 (10.0) | 0.058 |
|  | No | 134 | 39 (29.1) |  |  | 7 (5.2) |  |  | 21 (15.7) |  |  | 3 (2.2) |  |  | 3 (2.2) |  |  | 6 (4.5) |  |
| AIDS stages | |  |  |  |  |  |  |  |  |  |  |  |  |  |  |  |  |  |  |
|  | I-II | 137 | 31 (22.6) | 0.005 |  | 1 (0.7) | 0.002 |  | 26 (19.0) | 0.069 |  | 1 (0.7) | 0.065 |  | 0 | 0.054 |  | 4 (2.9) | 0.010 |
|  | III-IV | 247 | **90 (36.4)** |  |  | **23 (9.3)** |  |  | 30 (12.1) |  |  | 12 (4.9) |  |  | 7 (2.8) |  |  | **27 (10.9)** |  |

*Fisher’ s Exact Test. ^a^p value = MSM vs MSW. ^b^p value= MSM vs MSW vs Other. Bold=the values significant higher than that in the same group were shown in bold.
