# Supplementary material for: A baseline epidemiological study of the co-infection of enteric protozoans with human immunodeficiency virus among men who have sex with men from Northeast China
Source: PLoS Negl Trop Dis. 2022 Sep 6;16(9):e0010712. doi: 10.1371/journal.pntd.0010712 (PMC9447920; doi:10.1371/journal.pntd.0010712)
Supplement: S10 Table — (DOCX) [file pntd.0010712.s010.docx]

**S10 Table Parasite infection proportions among all HIV-positive participants with different diarrhea status**

| Diarrhea^#^ | | No. | Any of parasites | |  | *E. histolytica* | |  | *E. bieneusi* | |  | *Cryptosporidium* spp*.* | |  | *C. cayetanensis* | |  | *B. hominis* | |
| --- | --- | --- | --- | --- | --- | --- | --- | --- | --- | --- | --- | --- | --- | --- | --- | --- | --- | --- | --- |
|  |  |  | n (%) | p value |  | n (%) | p value |  | n (%) | p value |  | n (%) | p value |  | n (%) | p value |  | n (%) | p value |
| Yes | PD^a^ | 64 | **34 (53.1)** | 0.000 |  | **8 (12.5)** | 0.010 |  | 8 (12.5) | 0.476 |  | **5 (7.8)** | 0.003 |  | 3 (4.7) | 0.163 |  | 4 (6.3) | 1.000 |
|  | HD^b^ | 114 | **44 (38.6)** | 0.001 |  | 6 (5.3) | 0.607 |  | **21 (18.4)** | 0.022 |  | 3 (2.6) | 0.259 |  | 1 (0.9) | 1.000 |  | 11 (9.6) | 0.258 |
|  | Subtotal***^c^*** | 178 | **78 (43.8)** | 0.083 |  | 14 (7.9) | 0.144 |  | 29 (16.3) | 0.399 |  | 8 (4.5) | 0.221 |  | 4 (2.2) | 0.263 |  | 15 (8.4) | 0.587 |
| No | ND^d^ | 206 | 43 (20.9) | 0.000 |  | 7 (3.4) | 0.071 |  | 19 (9.2) | 0.044 |  | 1 (0.5) | 0.024 |  | 2 (1.0 | 0.553 |  | 12(5.8) | 0.424 |
| Total^e^ | | 384 | 121 (31.5) | 0.000 |  | 21 (5.5) | 0.055 |  | 48 (12.5) | 0.037 |  | 9 (2.3) | 0.024 |  | 6 (1.6) | 0.553 |  | 27 (7.0) | 0.321 |

PD=present diarrhea. HD=historic diarrhea, as described in the methods. ND=non-diarrhea. Bold=the values higher than that in the same group were shown in bold. ^#^Parasite infection identified as a single infection. ^a^p value =PD vs ND. ^b^p value =HD vs ND. ^c^p value=PD vs HD. ^d^p value=Yes vs No. ^e^p value =PD vs HD vs ND.
