# Supplementary material for: A baseline epidemiological study of the co-infection of enteric protozoans with human immunodeficiency virus among men who have sex with men from Northeast China
Source: PLoS Negl Trop Dis. 2022 Sep 6;16(9):e0010712. doi: 10.1371/journal.pntd.0010712 (PMC9447920; doi:10.1371/journal.pntd.0010712)
Supplement: S11 Table — (DOCX) [file pntd.0010712.s011.docx]

**S11 Table The association of enteric parasite infection with CD4^+^ T cell count of MSM HIV-positive participants**

| **CD4** | **n** | **Any of parasites** | |  | ***E. histolytica*** | |  | ***E. bieneusi*** | |  | ***Cryptosporidium* spp*.*** | |  | ***C. cayetanensis*** | |  | ***B. hominis*** | |
| --- | --- | --- | --- | --- | --- | --- | --- | --- | --- | --- | --- | --- | --- | --- | --- | --- | --- | --- |
|  |  | **n** (%) | **p value** |  | **n** (%) | **p value** |  | **n** (%) | **p value** |  | **n** (%) | **p value** |  | **n** (%) | **p value** |  | **n** (%) | **p value** |
| 0-150 | 121 | **47 (38.8)** | 0.022 |  | **13 (10.7)** | 0.027 |  | 16 (13.2) | 0.793 |  | **9 (7.4)** | 0.001^*^ |  | 3 (2.5) | 0.308^*^ |  | 10 (8.3) | 0.46 |
| 151-350 | 97 | 23 (23.7) |  |  | 3 (3.1) |  |  | 16 (16.5) |  |  | 0 |  |  | 0 |  |  | 5 (5.2) |  |
| >350 | 90 | 22 (24.4) |  |  | 3 (3.3) |  |  | 13 (14.4) |  |  | 0 |  |  | 2 (2.2) |  |  | 4 (4.4) |  |

^*^Fisher’s Exact Test. Bold=the values significant higher than that in the same group were shown in bold.
