# Supplementary material for: A baseline epidemiological study of the co-infection of enteric protozoans with human immunodeficiency virus among men who have sex with men from Northeast China
Source: PLoS Negl Trop Dis. 2022 Sep 6;16(9):e0010712. doi: 10.1371/journal.pntd.0010712 (PMC9447920; doi:10.1371/journal.pntd.0010712)
Supplement: S12 Table — (DOCX) [file pntd.0010712.s012.docx]

**S12 Table The association of parasite infection with the cluster groups**

| **Parasites** | | **Groups** | | |  | **p value** | |
| --- | --- | --- | --- | --- | --- | --- | --- |
|  |  | **Interest** | **Other** | **Uninfected** |  | **(Interest vs Other)** | **(Interest vs Uninfected)** |
| *E. bieneusi* | Group A | 7 | 20 | 67 |  | 0.0082 | 0.1299 |
|  | Group B | 28 | 18 | 126 |  |  |  |
| *Cryptosporidium spp.* | Group A | 3 | 24 | 67 |  | 0.7047 | 0.6973 |
|  | Group B | 4 | 42 | 126 |  |  |  |
| *E. histolytica* | Group A | 9 | 18 | 67 |  | 0.1302 | 0.1470 |
|  | Group B | 7 | 39 | 126 |  |  |  |
| *C.. cayetanensis* | Group A | 2 | 25 | 67 |  | 0.6234 | 0.6130 |
|  | Group B | 2 | 44 | 126 |  |  |  |
| *B. hominis* | Group A | 6 | 21 | 67 |  | 0.3090 | 0.2059 |
|  | Group B | 5 | 41 | 126 |  |  |  |
