# Supplementary material for: A baseline epidemiological study of the co-infection of enteric protozoans with human immunodeficiency virus among men who have sex with men from Northeast China
Source: PLoS Negl Trop Dis. 2022 Sep 6;16(9):e0010712. doi: 10.1371/journal.pntd.0010712 (PMC9447920; doi:10.1371/journal.pntd.0010712)
Supplement: S1 Fig — (DOCX) [file pntd.0010712.s013.docx]

**S1 Fig Phylogenetic relationship of *E. bieneusi genotypes* identified in the present study and known genotypes found in Heilongjiang Province, China, deposited in GenBank**

Phylogenetic relationships of *E. bieneusi* genotype groups. The relationship between *E. bieneusi* genotypes identified in the present study and other known genotypes isolated in different hosts in Heilongjiang Province, China, deposited in GenBank, were inferred by a neighbor-joining analysis of ITS sequences based on genetic distance by the Kimura 2-parameter model. The numbers on the branches are percent bootstrapping values from 1000 replicates. Each sequence is presented with its accession number and genotype designation. The circles filled in red are the representative of corresponding genotypes. The squares filled in red are the novel genotypes identified in this study. The squares filled in black are those found only in humans. The circles filled in black are those found in humans and animals. The open circles are those found in animals.
