# Supplementary material for: A baseline epidemiological study of the co-infection of enteric protozoans with human immunodeficiency virus among men who have sex with men from Northeast China
Source: PLoS Negl Trop Dis. 2022 Sep 6;16(9):e0010712. doi: 10.1371/journal.pntd.0010712 (PMC9447920; doi:10.1371/journal.pntd.0010712)
Supplement: S2 Fig — (DOCX) [file pntd.0010712.s014.docx]

**S2 Fig Phylogenetic relationship of gp60 subtypes of *C. meleagridis***


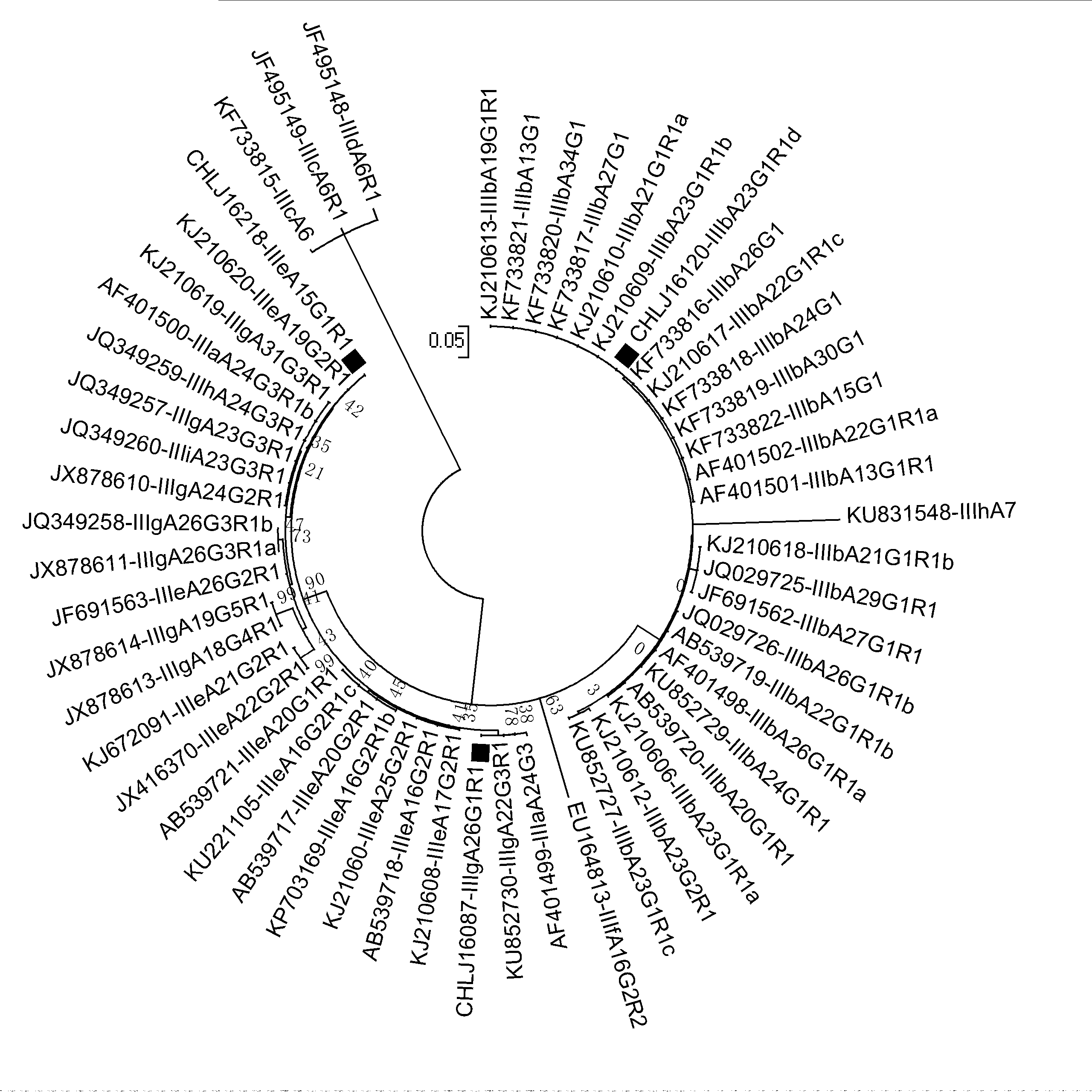


Phylogenetic relationship of gp60 subtypes of *C. meleagridis*. The relationships between *C. meleagridis* subtypes identified in the present study and known subtypes deposited in the GenBank were inferred by a neighbor-joining analysis of gp60 gene sequences based on genetic distance by the Kimura 2-parameter model. The numbers on the branches are percent bootstrapping values from 1000 replicates. Each sequence is presented with its accession number and subtype designation. The squares filled in black indicate the subtypes identified in this study.
